# Machine learning–based differentiation of major depressive disorder and bipolar disorder using entropy-derived EEG biomarkers in drug-naïve patients

**DOI:** 10.1017/S1092852926100923

**Published:** 2026-05-11

**Authors:** Hyeon-Ho Hwang, Kang-Min Choi, Hyeon-Ah Lee, Sungkean Kim, Ji Sun Kim

**Affiliations:** 1 Department of Human-Computer Interaction, Hanyang University, Ansan, Republic of Korea; 2 Department of Electronic Engineering, Seoul National University of Science and Technology (Seoultech), Seoul, Republic of Korea; 3 Department of Psychiatry, Soonchunhyang University Cheonan Hospital, Cheonan, Republic of Korea

**Keywords:** Electroencephalography, band-scale entropy, cross-sample entropy, major depressive disorder, bipolar disorder, machine learning

## Abstract

**Objective:**

Major depressive disorder (MDD) and bipolar disorder (BD) exhibit overlapping depressive presentations, making early differentiation difficult and misdiagnosis clinically consequential. Entropy-based resting-state electroencephalography (EEG) markers were evaluated for differentiating drug-naïve patients with stable MDD, converted BD, and diagnosed BD.

**Methods:**

Resting-state EEG was acquired from 92 drug-naïve patients: 50 with stable MDD, 16 who converted to bipolar II disorder over 3 years, and 26 with diagnosed BD. Band-scale entropy and cross-sample entropy (CSE) were analyzed. Group differences were tested by analysis of covariance adjusted for depressive severity; classification was evaluated by nested cross-validation with permutation testing.

**Results:**

Relative to stable MDD, diagnosed BD showed higher theta- and alpha-band entropy in frontal and central regions and lower values for selected intra- and inter-regional CSE measures. Two CSE measures correlated positively with non-planning impulsiveness in diagnosed BD. Converted BD showed intermediate values without significant pairwise differences. Prospective discrimination of stable MDD versus converted BD was modest (balanced accuracy, 65.4%; area under the receiver operating characteristic curve [ROC-AUC], 0.661). Classification was strongest for stable MDD versus diagnosed BD, yielding 78.9% pooled accuracy, 76.6% balanced accuracy, and an ROC-AUC of 0.832 when entropy features were combined with depressive severity; combined entropy outperformed either single-family configuration.

**Conclusions:**

Entropy-derived EEG measures differentiated stable MDD from diagnosed BD and suggested a weaker, intermediate profile in converted BD. They may serve as candidate adjunctive biomarkers for mood disorder differentiation, although larger prospective cohorts are required to clarify their value in predicting conversion to bipolar II disorder.

## Introduction

A notable proportion of patients initially presenting with a major depressive episode are subsequently rediagnosed with bipolar disorder (BD), a diagnostic conversion with profound clinical consequences. In a recent propensity score–matched cohort, 17.5% of adults aged 18–40 who eventually converted to BD experienced this transition within 3 years of their initial major depressive disorder (MDD) diagnosis,^1^ and such conversion is associated with greater illness burden, including more frequent depressive recurrences, higher rates of hospitalization and suicidal behavior, and different treatment requirements.^2^ Misdiagnosis of BD as MDD also poses direct therapeutic risks: long-term antidepressant treatment in BD has been associated with a substantially elevated risk of new manic or hypomanic episodes,^3^ and patients with unrecognized BD treated with antidepressants demonstrate markedly higher rates of suicide attempts compared with both recognized BD and non-bipolar patients.^4^ These findings underscore an urgent need for tools that can identify, at the earliest stage of illness, depressed patients likely to follow a bipolar trajectory.

Because clinical features alone are insufficient to reliably predict diagnostic conversion at initial presentation, neurophysiological biomarkers have garnered growing interest.^5^ Neuroimaging studies have identified structural and functional differences between established MDD and BD,^6^ and resting-state electroencephalography (EEG) has attracted particular attention owing to its high temporal resolution, non-invasiveness, and capacity to capture dynamic neural activity. 7, 8 However, most existing EEG studies have compared patients with confirmed diagnoses in cross-sectional designs, often including medicated patients whose brain activity may be confounded by pharmacological effects.^9^^,^^10^ Few investigations have examined whether EEG signatures recorded at initial depressive presentation—before diagnostic conversion has occurred—can prospectively distinguish future converters from patients who will remain within the MDD spectrum. This gap is significant because a predictive biomarker must be identifiable before the clinical outcome it aims to forecast.

To address these limitations, a prospective longitudinal design was adopted in which drug-naïve patients presenting with a depressive episode underwent resting-state EEG recording at their initial visit and were followed for approximately 3 years to ascertain the diagnostic outcome. Among 66 drug-naïve patients initially diagnosed with MDD, 16 were rediagnosed with bipolar II disorder (BD-II) during follow-up (the “converted BD” group), while 50 maintained their MDD diagnosis (the “stable MDD” group). A reference group of 26 drug-naïve patients diagnosed with BD at initial presentation (the “diagnosed BD” group) was included to determine whether baseline neural activity of future converters already resembles that of patients with an established bipolar diagnosis. This 3-group design enables identification of EEG features that prospectively differentiate future converters from stable MDD patients and evaluation of whether such features reflect a neurophysiological profile characteristic of BD.

To characterize neural dynamics across these groups, entropy-based EEG measures quantifying nonlinear complexity and regularity of scalp-recorded signals were employed. Neural complexity differs between psychiatric disorders, with evidence that patients with BD exhibit altered signal irregularity relative to schizophrenia, suggesting that entropy measures may capture diagnostically relevant neurophysiological differences.^11^ Band-scale entropy was utilized to estimate frequency-specific complexity across theta, alpha, beta, and gamma bands without signal distortions from band-pass filtering prior to entropy computation.^11^^,^^12^ Furthermore, cross-sample entropy (CSE) was employed to assess intra- and inter-regional neural interaction complexity.^13^ Machine-learning classification using a nested cross-validation protocol designed to prevent data leakage was additionally performed, and multiple classifier families were compared to ensure that findings were not dependent on a single algorithm.

The primary aim was to determine whether entropy-based resting-state EEG biomarkers recorded at initial presentation can prospectively differentiate drug-naïve patients with stable MDD from those who subsequently convert to BD-II. It was hypothesized that (1) the converted BD group would exhibit a neural complexity profile intermediate between the stable MDD and diagnosed BD groups, reflecting a latent bipolar diathesis detectable prior to clinical conversion and (2) machine learning classification based on entropy features would achieve meaningful discriminative accuracy. Identification of such prospective markers would represent a step toward objective, EEG-based tools for early detection of bipolar risk among patients presenting with depression. The remainder of the article is structured as follows: section “Related work” reviews related work, section “Materials and methods” details materials and methods, section “Results” presents results, section “Discussion” discusses findings, and section “Conclusion and future work” concludes with future work.

## Related work

Three complementary research lines address the challenge of differentiating MDD from BD: neuroimaging and EEG-based studies, entropy-based quantification of EEG signal complexity, and machine-learning classification frameworks. Each domain has yielded promising findings, yet important gaps persist.

Within neuroimaging, structural and functional differences between established MDD and BD have been documented,^6^ and resting-state functional connectivity (FC) analyses have shown that default-mode and frontoparietal network patterns differentiate the 2 disorders.^14^ A functional magnetic resonance imaging (fMRI) study using amygdala connectivity extended classification to an independent conversion sample,^15^ whereas a large-scale investigation using nested cross-validation found significant performance only for seed-based connectivity, suggesting that smaller studies may overestimate discriminative performance.^16^ EEG studies identified alpha-range alterations across psychiatric disorders and showed that coherence measures can aid MDD–BD differentiation.^17^^,^^18^ A systematic review further indicated that much of the resting-state EEG biomarker literature focused on MDD versus healthy controls and was marked by substantial population and methodological variability.^10^ Despite these advances, most differentiation studies remain cross-sectional and medication-exposed, and studies including patients who later converted from MDD to BD remain exceedingly rare.

Entropy-based measures offer a complementary approach beyond conventional spectral and connectivity analyses, with multiscale entropy (MSE) quantifying signal complexity across temporal scales and CSE providing a more stable assessment of inter-signal synchrony than earlier approximate-entropy-based estimators.^13^^,^^19^ Prior work showed frequency-specific band-scale entropy differences between schizophrenia and BD,^12^ and combining multiscale fuzzy entropy with relative power yielded significant classification accuracy between these groups.^11^ CSE also showed sensitivity to emotional state, with increased cross-signal regularity during distress.^20^ However, entropy-based differentiation of MDD and BD remains limited, and no previous study has combined band-scale entropy and CSE within a unified framework for mood disorder subtype discrimination in drug-naïve depressive cohorts.

Machine learning classification has been employed to assess the diagnostic utility of neurophysiological and entropy-derived features for mood disorder differentiation, with reported performance varying substantially across imaging modalities and validation schemes.^15^^,^^16^^,^^18^ In the entropy domain, classification based on complexity-derived features achieved significant schizophrenia–BD discrimination,^11^ but comparable entropy-based frameworks for MDD–BD classification remain scarce. Methodological weaknesses are also common: feature selection performed outside cross-validation can introduce selection bias, permutation testing has often been omitted, class imbalance has been handled inconsistently, and ablation, interpretability, and random-seed robustness analyses have rarely been evaluated together. Rigorous, fully integrated classification pipelines therefore remain uncommon in this literature.

In summary, the literature remains limited by the scarcity of prospective cohorts including patients later converting from MDD to BD, the predominance of medicated samples, the lack of integrated band-scale entropy and CSE analyses for mood disorder subtype differentiation, and inconsistent rigor in classification pipelines. These gaps support the need for prospective evaluation in a drug-naïve depressive cohort using integrated entropy measures and rigorously validated classification procedures. The methodological details are described in the following section.

## Materials and methods

### Participants

Participants were recruited from the Department of Psychiatry at Soonchunhyang University Cheonan Hospital in Korea between December 2018 and August 2021. The study included 3 groups: patients who maintained an MDD diagnosis throughout an approximately 3-year follow-up period (stable MDD group, *n* = 50), those who transitioned from MDD to BD-II within the same period (converted BD group, *n* = 16), and those with an established BD diagnosis at enrollment (diagnosed BD group, *n* = 26). The diagnosed BD group consisted of 7 participants with bipolar I disorder (BD-I) and 19 with BD-II; all patients in this group experienced a depressive episode at the time of EEG recording. The diagnostic conversion from MDD to BD-II in the converted BD group was confirmed based on the Diagnostic and Statistical Manual of Mental Disorders, Fifth Edition (DSM-5) criteria for hypomanic episodes.^21^ The DSM-5 stipulates that “A hypomanic episode that emerges during antidepressant treatment and persists at a fully syndromal level beyond the physiological effect of that treatment is sufficient evidence for a hypomanic episode and a diagnosis of bipolar II disorder.”^21^^,^^22^ All BD and MDD diagnoses were established by a board-certified psychiatrist in accordance with the DSM-5.^21^ Patients with a history of neurological disorders or other severe medical conditions were excluded during the initial screening interviews. The Institutional Review Board (IRB) and Ethics Committee of Soonchunhyang University Cheonan Hospital granted approval for this study (IRB no. 2018-10-032). All procedures were conducted in accordance with the approved guidelines, and all participants provided informed consent.^23^

### Psychological measures

Anxiety, depression, and impulsivity were assessed using 3 self-report instruments: the State–Trait Anxiety Inventory (STAI),^24^ the Beck Depression Inventory (BDI),^25^ and the Barratt Impulsiveness Scale, version 11 (BIS-11),^26^ respectively. The STAI quantifies the severity of state and trait anxiety, while the BDI measures the severity of depression. The BIS-11 assesses impulsivity as a personality trait, yielding an overall score (BIS-11 total score) and 3 subscale scores: motor impulsiveness (BIS-motor), non-planning impulsiveness (BIS-nonplan), and attentional impulsiveness (BIS-attentional).

### EEG acquisition

Resting-state EEG data were collected during the initial hospital visit of each participant. Participants were seated in a sound-attenuated room and asked to keep their eyes open for 5 min. EEG recordings were performed using a NeuroScan SynAmps2 amplifier (Compumedics USA, Charlotte, NC) and 62 Ag/AgCl electrodes arranged according to the extended 10–20 system and mounted on a Quik-Cap. Electrooculogram electrodes were positioned at the outer corners of each eye and above and below the left eye to capture horizontal and vertical movements, respectively. The reference electrodes were placed on both mastoids, and the ground electrode was positioned on the forehead. The EEG signals were sampled at 1000 Hz, band-pass filtered between 0.1 and 100 Hz, and notch filtered at 60 Hz to eliminate power line noise. Electrode impedance was maintained below 5 kΩ throughout the recording.

### EEG preprocessing

First, baseline correction was performed by subtracting the mean amplitude of each channel from the corresponding EEG signal to remove the direct current offset.^27^ Next, the artifact subspace reconstruction (ASR) algorithm was applied to attenuate high-amplitude transient artifacts. ASR operates by first identifying artifact-free calibration segments within the recording and estimating their statistical distribution. Following this calibration, continuous EEG data were segmented into 500-ms sliding windows and decomposed into subspaces using principal component analysis. Within each window, any principal component whose variance exceeded 20 standard deviations relative to the calibration distribution was identified as artifact-contaminated and reconstructed using the spatial structure of the clean reference data. Corrupted segments were thereby corrected in place rather than rejected, effectively attenuating transient artifacts while preserving the underlying neural activity.^28^

Following ASR, a wavelet-based artifact removal approach was applied.^29^ Each channel was decomposed using a 10-level discrete wavelet transform with a biorthogonal 4.4 wavelet basis.^11^ Level-dependent noise estimates were obtained through Bayesian estimation, and a soft thresholding rule was applied to separate artifact-related wavelet coefficients from neural components.^30^^,^^31^ The isolated artifact-related coefficients were reconstructed into a time-domain artifact signal via the inverse wavelet transform.^30^^,^^31^ This artifact signal was subsequently subtracted from the recording to eliminate artifact components while retaining neural activity.

After wavelet denoising, independent component analysis (ICA) was performed using the extended Infomax algorithm to identify and remove residual artifacts.^32^ Component classification was conducted using an automated classifier trained on a large independent component database, which assigned probability estimates for each independent component across predefined categories (brain, muscle, eye, heart, line noise, channel noise, and other).^33^ Components were rejected if the estimated probability of muscle artifact exceeded 0.50 or if the estimated probability of any other non-brain category exceeded 0.90.^27^ To preserve neural information, the maximum number of rejected components was constrained to 20% of the total independent components per participant (i.e., a maximum of 12 out of 62 components).^34^ Once artifact components had been removed, a common average reference was applied across all channels to standardize the voltage reference, completing the artifact removal phase.^35^ Importantly, the entire preprocessing pipeline described above—including ASR, wavelet denoising, ICA decomposition, and component classification—was executed as a fully automated procedure, thereby ensuring that all artifact rejection decisions were made without knowledge of diagnostic group membership.

For each participant, preprocessed continuous EEG recordings were divided into non-overlapping 30-s candidate segments. A segment was initially deemed artifact-free if the maximum absolute amplitude across all channels did not exceed 90 μV.^36^ Each candidate segment was further evaluated using a multi-metric quality control procedure that assessed peak-to-peak amplitude, channel-wise variance homogeneity, kurtosis, spectral slope, and temporal gradient stability; only segments that passed all criteria were retained for subsequent analysis.

The retained segments were then processed differently depending on the target entropy measure. For band-scale entropy analysis, each retained 30-s segment was subjected to MSE computation without further segmentation, and the subject-level band-scale entropy value for each feature was determined as the median across all retained segments. No additional frequency-domain filtering was applied prior to this computation, as the coarse-graining process inherent in MSE analysis functions as a low-pass filter.^37^ In contrast, for CSE analysis, each 30-s segment was divided into 15 non-overlapping epochs of 2 s each; the CSE was computed for each epoch, and the segment-level CSE value was determined as the median across the 15 epochs. Subject-level CSE values were likewise obtained by computing the median across all retained segments.

To assess robustness to segment selection, the sensitivity of entropy values to varying numbers of these segments was evaluated through repeated random sampling from the pool of eligible data. Specifically, varying subsamples were randomly drawn across multiple iterations, and the resulting entropy estimates were compared to quantify their stability as a function of the number of segments included. All preprocessing, segmentation, and entropy-robustness procedures described in this section were implemented in MATLAB R2021b (MathWorks, Natick, MA, USA); classifier robustness analyses are described separately in section “Feature selection and classification”.

### Band-scale entropy analysis

Band-scale entropy was employed to assess the complexity of the EEG data.^12^ This measure is derived from MSE analysis, which computes sample entropy (SampEn) across multiple temporal scales.^19^ The first step of MSE analysis involves generating a coarse-grained time series from the original EEG signal.^19^ For an original EEG series *x* of length *N*, a coarse-grained time series $y^{\tau }$ is generated for each scale factor *τ*, with each element $y_{i}^{\tau }$ calculated as follows:^19^

(1) $$y_{i}^{\tau }=\frac{1}{\tau }\sum_{n=\tau \left(i-1\right)+1}^{\mathrm{\tau i}}x_{n},1\leq i\leq \lfloor \frac{N}{\tau }\rfloor =L$$

In this equation, *L* denotes the length of the coarse-grained series, and *i* represents the index of its elements. For example, when *τ* = 1, the time series $y^{1}$ is identical to the original series *x.* The present analysis employed a total of 70 scale factors, resulting in the shortest coarse-grained time series (corresponding to the largest *τ* value) containing 428 data points. This sample size is considered sufficient for reliable SampEn estimation, given the general recommendation for a time series length of $10^{m}$ to $20^{m}$ points, where *m* is the embedding dimension (set to 2 in this study; see below).^38^

Subsequently, the SampEn values for each coarse-grained time series $y^{\tau }$ were computed, denoted as $MSE\left(\tau \right)=SampEn\left(y^{\tau },\;m,\;r\right)$ (2).^19^ SampEn is defined as the negative natural logarithm of the conditional probability that 2 sequences of *m* consecutive data points that are similar within tolerance *r* also remain similar when extended by one data point (to length *m* + 1).^19^ Sequence similarity was assessed using the Chebyshev distance, with parameters set to the commonly used values of *m* = 2 and *r* = 0.15 × the standard deviation of each time series.^12,19^ Once SampEn was obtained at each scale factor, region-level measures were derived by averaging the MSE(*τ*) values across electrodes within each of 6 electrode clusters, with each cluster representing a distinct brain region.^12^ These clusters included left frontal (AF3, F1, F3, and F5), right frontal (AF4, F2, F4, and F6), left central (FC1, FC3, FC5, C1, C3, C5, CP1, CP3, and CP5), right central (FC2, FC4, FC6, C2, C4, C6, CP2, CP4, and CP6), left parietal (P1, P3, P5, PO3, and PO5), and right parietal (P2, P4, P6, PO4, and PO6).^11^ This process yielded an MSE curve for each region.

The definition of frequency-band-specific scale factor ranges was grounded in previous research demonstrating that lower scale factors reflect the complexity of higher-frequency components, whereas higher scale factors reflect that of lower-frequency components.^11^, ^12^ Specifically, prior studies found that the MSE curves for simulated sinusoidal rhythms of 50, 30, 12, and 8 Hz peaked at scale factors 7, 13, 30, and 41, respectively.^12^ Based on these findings, scale factor ranges were defined for the following frequency bands: gamma (scale factors 7–12), beta (scale factors 13–29), alpha (scale factors 30–40), and theta (scale factors 41–70).^12^ The band-scale entropy for each brain region and frequency band was then calculated by averaging the MSE(*τ*) values within the corresponding range of scale factors.^12^

### Intra- and inter-regional cross-sample entropy analysis

CSE is a refinement of cross-approximate entropy that exhibits improved statistical consistency across varying data lengths and parameter settings.^13^ This measure quantifies the degree of asynchrony between 2 distinct yet related time series by assessing the regularity of their cross-recurrence patterns.^13^ Given 2 sequences of vectors, $X_{i}^{\left(m\right)}=\left\{x_{i},x_{i+1},\ldots ,x_{i+m-1}\right\}\;\text{and}\;Y_{j}^{\left(m\right)}=\left\{y_{j},y_{j+1},\ldots ,y_{j+m-1}\right\}$ , where 1≤i,j≤N−m , the CSE can be calculated as follows:^13^

(3) $$CSE\left(m,\;r,\;N\right)=-\ln\frac{\sum_{i=1}^{N-m}n_{i}^{\left(m+1\right)}}{\sum_{i=1}^{N-m}n_{i}^{\left(m\right)}}$$

In this formula, $n_{i}^{\left(m\right)}$ represents the number of template matches obtained by comparing vector $X_{i}^{\left(m\right)}$ from time series *X* with all vectors $Y_{j}^{\left(m\right)}$ from time series *Y.* ^13^ A template match occurs between $X_{i}^{\left(m\right)}$ and $Y_{j}^{\left(m\right)}$ when the Chebyshev distance between them is less than or equal to the threshold *r.* ^13^ Correspondingly, $n_{i}^{\left(m+1\right)}$ represents the number of template matches found when comparing vectors of length *m* + 1 using the same criterion.^13^

The natural logarithm transforms the ratio of template matches into an entropy measure. Lower CSE values indicate greater temporal synchrony between time series, reflecting more predictable cross-signal interactions.^13^ Based on these properties, the CSE analysis in the present study was performed on the same 6 brain regions defined in the MSE analysis, with parameters *m* = 2 and *r* = 0.15, within commonly recommended ranges for (cross-) SampEn-type estimators.^39^ Before the CSE was calculated, each time series was normalized using a z-score transformation to achieve a mean of zero and a standard deviation of one. On the basis of the normalized signals, 2 distinct CSE measures were computed to assess neural synchrony at multiple spatial scales: intra-regional CSE and inter-regional CSE. Intra-regional CSE quantified the synchrony among electrodes within the same brain region and was calculated as the mean CSE across all unique electrode pairs within each region, excluding self-pairs. As a complementary measure, inter-regional CSE quantified synchrony between different brain regions and was calculated as the mean CSE across all unique electrode pairs connecting 2 distinct regions.

### Statistical analysis

Group differences in EEG entropy measures were evaluated across the 3 diagnostic groups (stable MDD, converted BD, and diagnosed BD) using analysis of covariance (ANCOVA), with BDI score included as a continuous covariate to control for depression severity. BDI scores were mean-centered to aid interpretation of covariate-adjusted group means.^40^ The feature set comprised 45 EEG-derived measures, consisting of 24 band-scale entropy features and 21 CSE features. For each feature, an ANCOVA model was fitted with diagnostic group as a 3-level categorical factor and mean-centered BDI as a continuous covariate. The omnibus group effect was tested using an *F* statistic comparing a full model (intercept, 2 group indicators with stable MDD as the reference category, and mean-centered BDI) with a reduced model containing only the intercept and mean-centered BDI (numerator degrees of freedom = 2; denominator degrees of freedom = *N* − 4).

To account for unequal sample sizes, potential heteroscedasticity, and residual non-normality without relying on strict parametric assumptions, inferential *p*-values were obtained using a restricted-residual wild bootstrap approach.^41^ Under the null hypothesis of no group effect, residuals from the reduced model were multiplied by independently drawn wild weights from the Mammen 2-point distribution and added to the reduced-model fitted values to generate bootstrap response vectors.^41^^,^^42^ For each replicate, both reduced and full models were refitted and feature-wise *F* statistics were recomputed.^41^^,^^42^ A total of 20,000 replicates (*B*) were generated with a fixed random seed. Upper-tail *p*-values were computed with a + 1 correction, (1 + count)/ (1 + *B*), to avoid zero *p*-values.^43^

The primary analysis was a global set-level test across all 45 entropy features. The global test statistic was defined as the sum of the feature-wise *F* statistics, and the global *p*-value was estimated from the bootstrap distribution of this sum statistic.^41^ Secondary (exploratory) analyses were conducted at the feature level by reporting wild bootstrap *p*-values for the omnibus group effect, partial eta squared (*η*^2^ₚ) as an effect-size index, and covariate-adjusted group means evaluated at the sample mean BDI level.^44,45^

For features showing a significant omnibus group effect at the feature level (*p* < 0.05), exploratory post hoc comparisons were conducted using 3 prespecified linear contrasts (converted BD versus stable MDD, diagnosed BD versus stable MDD, and diagnosed BD versus converted BD). Contrast-specific *p*-values were obtained from the wild bootstrap distribution of the corresponding *t* statistics using 2-sided testing based on absolute values.^41,42^ The Holm–Bonferroni method was applied to correct for multiple comparisons across the 3 contrasts within each feature.^46^

To examine the clinical relevance of group-discriminating entropy features, within-group Spearman rank correlation analyses were conducted for features showing significant post hoc group differences after Holm–Bonferroni correction.^47^ For each such feature, the analysis was performed separately within each group involved in the significant contrast. Each selected entropy feature was correlated with 7 psychological measures: state anxiety and trait anxiety from the STAI, depression severity from the BDI, and BIS-attentional, BIS-motor, BIS-nonplan, and BIS-11 total score from the BIS-11. The Spearman rank correlation coefficient (*ρ*) was used as the effect-size measure.^47^

Inferential *p*-values for each feature–clinical-measure pair were obtained using a restricted-residual wild bootstrap procedure applied to rank-transformed variables.^41^^,^^48^ Both the entropy feature and the clinical variable were rank-transformed, and a simple linear regression of the ranked feature on the ranked clinical variable was fitted.^48^ Under the null hypothesis of no association, residuals from an intercept-only reduced model were multiplied by wild weights drawn from the Mammen 2-point distribution and added to the reduced-model fitted values; the full model was then refitted and the slope *t* statistic was recomputed for each replicate.^41^^,^^42^ A total of 20,000 replicates were generated with a fixed random seed, and 2-sided *p*-values were computed with the +1 correction.^43^ A global test across all feature–clinical-measure pairs within each group was conducted using the sum of squared *t* statistics as the global test statistic, with the global *p*-value estimated from the bootstrap distribution of this sum.^41^ Within each feature, Holm–Bonferroni correction was applied across the 7 clinical scales.^46^

As a supplementary sensitivity analysis, the stability of feature-level group separation between stable MDD and diagnosed BD under varying segment counts was assessed using unadjusted Mann–Whitney *U* tests. For each segment count *k* (ranging from 1 to 8), *k* segments were randomly drawn without replacement from the pool of retained artifact-free 30-s segments for each participant across 100 iterations; the subject-level entropy value for each feature at each iteration was defined as the median across the sampled segments. At each *k* and iteration, 2-sided Mann–Whitney *U* tests were performed separately for each of the entropy features that showed significant covariate-adjusted post hoc group differences in the primary ANCOVA. These tests were conducted without covariate adjustment to provide a conservative, model-free assessment of whether raw group separation persisted across segment-selection conditions. Stability was quantified as the proportion of iterations in which unadjusted significance (*p* < 0.05) was observed at each *k.* All statistical analyses described in this section were performed using MATLAB R2021b.

### Feature selection and classification

Machine-learning analyses were performed using the full cohort (total *n* = 92) to evaluate the diagnostic classification utility of entropy-derived EEG biomarkers under class imbalance. Three binary classification tasks were evaluated with task-specific sample sizes, as described below. First, stable MDD was compared with the BD spectrum (*n* = 92), where the converted BD and diagnosed BD groups were combined into a single positive class. Second, stable MDD was compared with converted BD (*n* = 66), with the diagnosed BD group (*n* = 26) excluded. Third, stable MDD was compared with diagnosed BD (*n* = 76), with the converted BD group (*n* = 16) excluded.

The predictor set comprised 45 EEG variables derived from band-scale entropy (6 regions × 4 frequency bands) and intra- and inter-regional CSE. To examine the contribution of depressive symptom severity, 3 feature configurations were defined for the first and third tasks (stable MDD versus BD spectrum; stable MDD versus diagnosed BD): EEG-only, EEG + BDI, and BDI-only. Because BDI scores in these tasks spanned clinically distinct ranges across diagnostic groups, the inclusion of BDI was motivated by its potential role as a complementary clinical predictor. For the second task (stable MDD versus converted BD), only the EEG-only configuration was evaluated; BDI was omitted a priori on the basis that both groups presented with comparable depressive symptom severity at the time of EEG recording, limiting its expected discriminative value. Feature configuration comparisons in the first and third tasks were conducted entirely within the nested cross-validation framework described below.

Model development and evaluation were conducted using nested cross-validation designed to prevent data leakage.^49,50^ Within each cross-validation split, features were standardized to zero mean and unit variance; feature selection, class-imbalance handling, decision-threshold determination, and classifier fitting were then performed exclusively on training data and applied to the corresponding test data.^51,52^ Hyperparameter optimization was conducted in the inner loop using stratified 5-fold cross-validation, and generalization performance was estimated in the outer loop using repeated stratified *k*-fold procedures (5 folds × 20 repeats).^50^ Hyperparameters were optimized by randomized search (60 iterations) using the area under the receiver operating characteristic curve (ROC-AUC) as the primary optimization criterion.^53^ To ensure reproducibility, random seeds were fixed across all stochastic operations.

Class imbalance was addressed within the training data via class-balanced learning (class-weighted losses for classifiers supporting this mechanism) and resampling-based balancing,^54,55^ including synthetic minority over-sampling technique (SMOTE) and borderline-SMOTE.^56,57^ The option of retaining the original class distribution without resampling was also included as a candidate within the hyperparameter search. Resampling was confined to training partitions both during inner-loop tuning and during refitting on the outer training data, to prevent data leakage.^52^

Feature selection was embedded within the cross-validation pipeline as a single step positioned before resampling. Three candidate strategies were compared as mutually exclusive alternatives within the hyperparameter search: no feature selection (i.e., retaining the complete feature set); a univariate filter-based approach using analysis of variance (ANOVA) *F*-statistics to rank features by linear association with the target class (with the top-*k* features retained, where *k* was optimized within the inner cross-validation);^58^ and wrapper-based selection using recursive feature elimination (RFE) with a logistic regression estimator.^59^ This wrapper method iteratively pruned 10% of features per step based on the absolute magnitude of model coefficients until reaching a target number of features (also optimized within the inner cross-validation).^60^ Across all strategies, the selection method and its associated hyperparameters were jointly optimized within the inner cross-validation to prevent selection bias.^49,51^ Following feature selection and resampling, 7 classifier families were compared: support vector machine with a radial basis function (RBF) kernel (RBF-SVM), linear SVM,^61^ regularized logistic regression (L1/L2),^62,63^ ridge classifier,^64^ linear discriminant analysis,^65^ random forest,^66^ and multilayer perceptron (MLP).^67^

Performance metrics (accuracy, balanced accuracy, sensitivity, specificity, and F1-score) were computed for each outer fold and summarized as means and standard deviations to characterize fold-level variability.^68^ In addition, pooled subject-level out-of-fold predictions were aggregated across all outer folds and repeats to derive pooled performance estimates, which served as the primary measure of overall generalization.^50^ Receiver operating characteristic (ROC) curves and the area under the ROC curve (ROC-AUC) were calculated using these pooled out-of-fold decision scores.^69^ Bootstrap 95% confidence intervals (2000 iterations) were estimated for all pooled performance metrics to quantify uncertainty.^70^ Separately, classification thresholds were determined on training data by maximizing the Youden *J* statistic within the nested procedure to avoid reliance on a fixed default threshold.^71^ The derived thresholds were then applied to the corresponding outer test folds.

Permutation testing was performed for the model family selected by composite mean-rank aggregation across 6 pooled performance metrics (balanced accuracy, ROC-AUC, accuracy, sensitivity, specificity, and F1-score). Balanced accuracy was used as the primary metric for permutation inference, and the complete nested cross-validation procedure—including hyperparameter optimization in the inner loop—was re-executed for each of 200 random label permutations to generate its null distribution.^72^ For each permuted evaluation, decision thresholds were re-determined from the corresponding outer training partition by maximizing the Youden *J* statistic. A one-sided permutation *p*-value was computed as the proportion of permutation scores greater than or equal to the observed mean score (with a plus-one adjustment).^43^

Ablation analyses were performed under the same nested evaluation framework to characterize the contribution of entropy feature families. Band-scale entropy, CSE, and the combined feature set were evaluated, with BDI score integrated into each feature configuration for the first and third tasks, in which BDI was included as a complementary clinical predictor in the main analysis. In addition to feature-family ablation, robustness checks were conducted to assess the stability of the results. Sensitivity to segment choice was quantified by re-evaluating the best-performing model under repeated random sampling from the pool of preprocessed, artifact-free, non-overlapping 30-s segments. This procedure was repeated for 100 iterations, and the resulting performance distribution was summarized. Finally, robustness to randomization was assessed by repeating the full analysis across 10 distinct random seeds (0–9) and aggregating performance across seeds.

Model interpretability was evaluated using Shapley Additive Explanations (SHAP) values computed for the best-performing tuned model to characterize global feature contributions.^73^For reproducibility, all machine learning analyses described in this section—from nested cross-validation through interpretability assessment—were implemented in Python (version 3.11.14) using scikit-learn (version 1.8.0), imbalanced-learn (version 0.14.1), and SHAP (version 0.50.0).^73–75^

## Results

### Demographic and psychological measures

Demographic, clinical, and EEG recording characteristics for the stable MDD (*n* = 50), converted BD (*n* = 16), and diagnosed BD (*n* = 26) cohorts are summarized in Table 1. No significant group differences were observed in sex distribution, age, educational attainment, state anxiety (STAI-S), trait anxiety (STAI-T), BIS-motor, BIS-nonplan, BIS-attentional, BIS-11 total score, or the number of artifact-free 30-second EEG segments per participant (all *p* ≥ 0.101). BDI scores differed significantly across groups (*p* = 0.001): post hoc comparisons indicated that the diagnosed BD group (35.27 ± 16.45) scored significantly lower than both the converted BD group (48.50 ± 10.53) and the stable MDD group (49.60 ± 10.53). BDI was therefore included as a covariate in subsequent EEG entropy analyses.

**Table 1.** Demographic, Clinical, and EEG Recording Characteristics of the Study Participants Table 1. long description.

Variable	Stable MDD^a^	Converted BD^b^	Diagnosed BD^c^	P	Post hoc
	(*n* = 50)	(*n* = 16)	(*n* = 26)		
Sex (male/female)	14/36	5/11	11/15	0.447	
Age (years)	27.64 ± 6.99	24.44 ± 4.72	31.15 ± 10.41	0.101	
Education (years)	13.50 ± 2.26	13.25 ± 1.77	13.62 ± 1.96	0.749	
STAI					
State anxiety (STAI-S)	63.38 ± 9.96	61.19 ± 7.83	58.92 ± 11.00	0.180	
Trait anxiety (STAI-T)	63.64 ± 8.78	63.94 ± 8.19	58.58 ± 11.42	0.145	
BDI	49.60 ± 10.53	48.50 ± 10.53	35.27 ± 16.45	0.001	a > c b > c
BIS–11					
BIS-motor	29.56 ± 5.71	28.81 ± 3.76	29.15 ± 3.28	0.942	
BIS-nonplan	25.68 ± 4.82	23.88 ± 3.61	25.23 ± 3.72	0.355	
BIS-attentional	20.94 ± 4.39	21.00 ± 3.81	20.81 ± 3.06	0.810	
BIS–11 total score	76.18 ± 12.49	73.69 ± 8.84	75.19 ± 7.89	0.842	
EEG recording					
No. of artifact-free 30-s segments	8.40 ± 2.55	8.62 ± 2.16	9.00 ± 2.87	0.176	

*Note:* Continuous variables are presented as mean ± SD; categorical variables are presented as counts. *P*-values are from χ^2^ test, one-way ANOVA, Welch’s ANOVA, or Kruskal–Wallis test, selected according to data type and distributional properties. Significant omnibus tests were followed by pairwise Welch *t*-tests.

Abbreviations: MDD, major depressive disorder; BD, bipolar disorder; BD-I, bipolar I disorder; BD-II, bipolar II disorder; STAI, State–Trait Anxiety Inventory; STAI-S, STAI State Anxiety subscale; STAI-T, STAI Trait Anxiety subscale; BDI, Beck Depression Inventory; BIS-11, Barratt Impulsiveness Scale, version 11; BIS-motor, Motor Impulsiveness; BIS-nonplan, Non-Planning Impulsiveness; BIS-attentional, Attentional Impulsiveness; No., number; SD, standard deviation.

a Stable MDD group.

b Converted BD group (all BD-II).

c Diagnosed BD group (BD-I: *n* = 7; BD-II: *n* = 19).

### Entropy-based EEG measures for mood disorder differentiation

**Figure 1.**
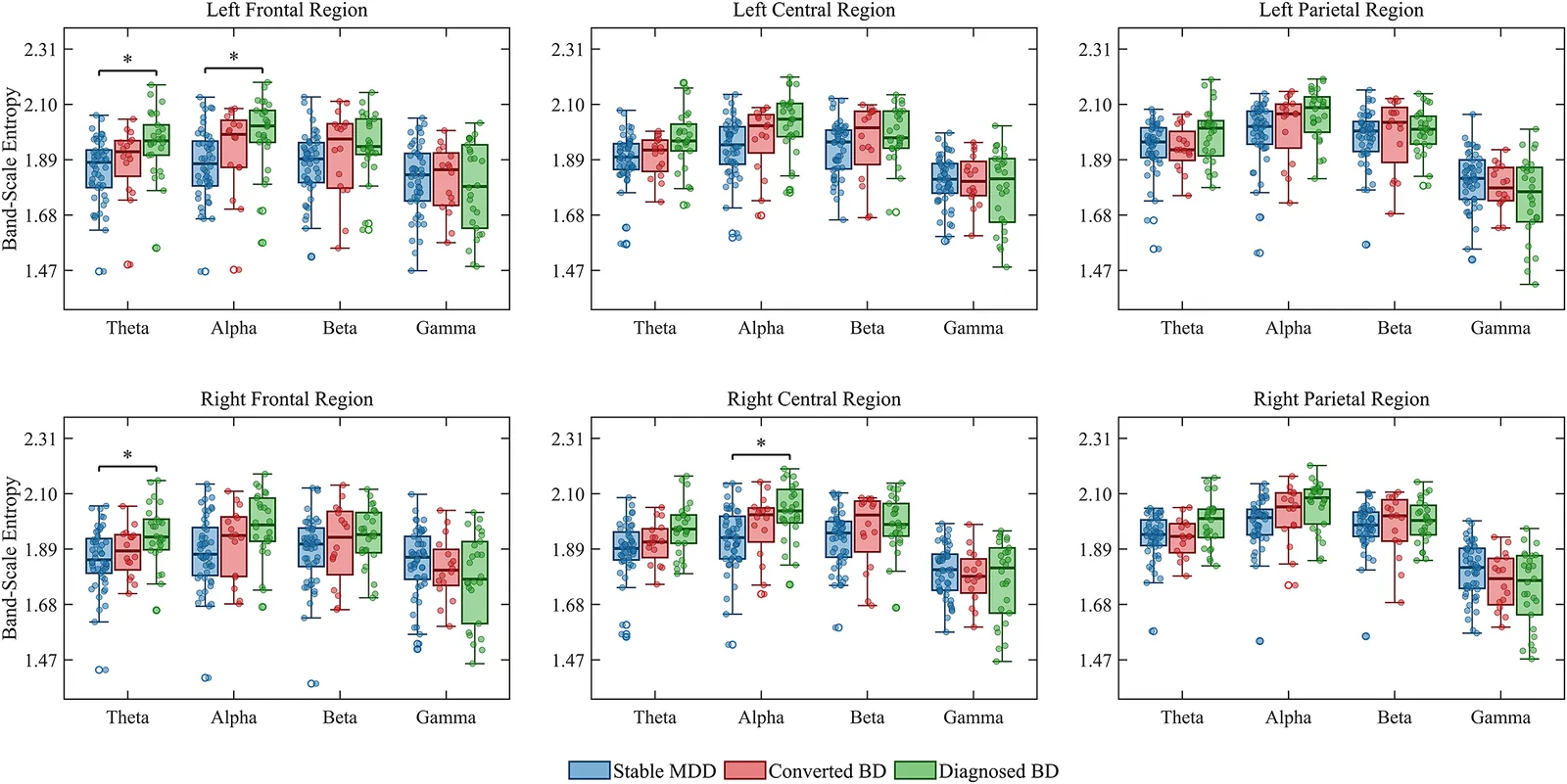
Band-scale entropy differs between stable major depressive disorder and diagnosed bipolar disorder across frontal and central brain regions. Each panel displays grouped box plots with overlaid individual data points for the stable MDD (blue; *n* = 50), converted BD (red; *n* = 16), and diagnosed BD (green; *n* = 26) groups across 4 frequency bands (theta, alpha, beta, and gamma) within 6 brain regions arranged in a 2 × 3 layout: left frontal, left central, and left parietal (top row) and right frontal, right central, and right parietal (bottom row). Boxes span the interquartile range (IQR; 25th–75th percentiles), horizontal lines within boxes indicate medians, and whiskers extend to the most extreme data points within 1.5 × IQR. Individual observations are shown as jittered scatter points. Brackets with asterisks (*) denote significant post hoc pairwise differences between the stable MDD and diagnosed BD groups (Holm–Bonferroni-corrected *p* < 0.05), identified in left frontal theta-band entropy (*p* = 0.030), left frontal alpha-band entropy (*p* = 0.031), right frontal theta-band entropy (*p* = 0.031), and right central alpha-band entropy (*p* = 0.037). Group differences were assessed using analysis of covariance with Beck Depression Inventory score as a covariate, and inferential *p*-values were obtained via a restricted-residual wild bootstrap procedure (20 000 replicates); post hoc contrasts were corrected using the Holm–Bonferroni method. Figure 1. long description.

The global set-level test across all 45 entropy features (24 band-scale entropy and 21 CSE) showed a significant omnibus group effect (sum-*F* = 173.73, *p* = 0.026; 20 000 wild bootstrap replicates with Mammen weights) after adjustment for mean-centered BDI. Band-scale entropy showed significant omnibus effects for 9 of 24 features, concentrated in the theta (5/6 regions) and alpha (4/6 regions) bands, with no significant effects in the beta or gamma bands. Across these features, covariate-adjusted means were highest in diagnosed BD, lowest in stable MDD, and intermediate in converted BD. Exploratory post hoc tests with Holm–Bonferroni correction showed higher entropy in diagnosed BD than in stable MDD for right frontal theta (*F*(2, 88) = 5.68, *η*^2^ₚ = 0.114, *p* = 0.016; *p*_Holm = 0.031), left frontal theta (*F*(2, 88) = 5.49, *η*^2^ₚ = 0.111, *p* = 0.018; *p*_Holm = 0.030), right central alpha (*F*(2, 88) = 5.52, *η*^2^ₚ = 0.112, *p* = 0.019; *p*_Holm = 0.037), and left frontal alpha (*F*(2, 88) = 5.34, *η*^2^ₚ = 0.108, *p* = 0.021; *p*_Holm = 0.031) (Figure 1). No other corrected pairwise contrasts, including those involving converted BD, reached significance.

**Figure 2.**
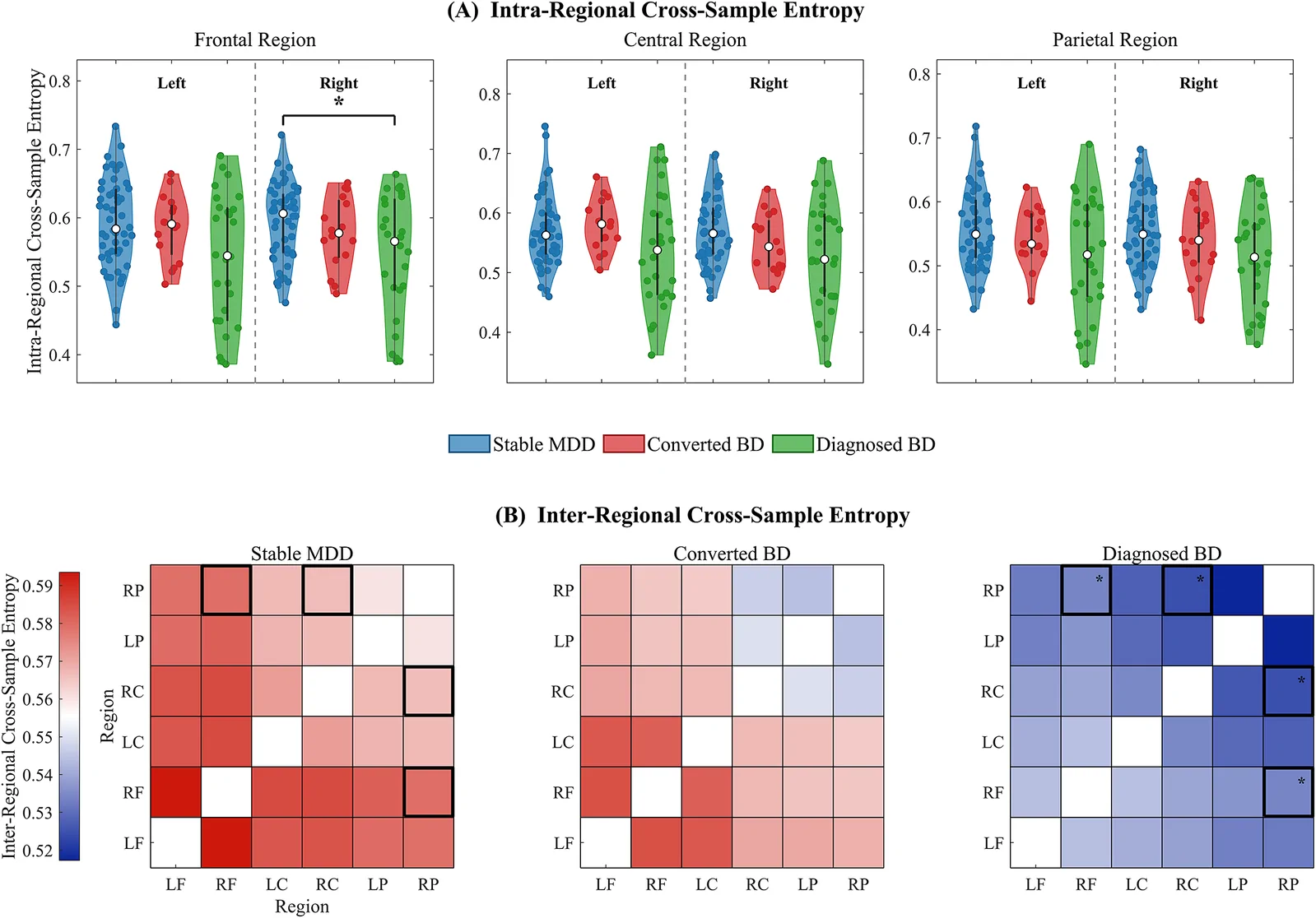
Cross-sample entropy measures across stable MDD, converted BD, and diagnosed BD groups. (A) Violin plots display the distribution of intra-regional cross-sample entropy (CSE) values across frontal, central, and parietal regions. Within each panel, distributions for the left and right hemispheres are separated by a vertical dashed line. Three study groups are represented: patients with stable major depressive disorder (stable MDD; blue, *n* = 50), patients who later converted to bipolar II disorder (converted BD; red, *n* = 16), and patients with bipolar disorder diagnosed at enrollment (diagnosed BD; green, *n* = 26). Each violin depicts the kernel density estimate, with individual data points overlaid. White circles denote medians, thick black bars represent interquartile ranges, and thin vertical lines indicate whiskers. The bracket with an asterisk (*) in the right frontal region denotes a significant post hoc difference between the stable MDD and diagnosed BD groups (Holm–Bonferroni-corrected *p* = 0.049). (B) Heatmaps depict group-mean inter-regional CSE values for each pairwise combination of 6 brain regions: left frontal (LF), right frontal (RF), left central (LC), right central (RC), left parietal (LP), and right parietal (RP). Diagonal entries (intra-regional pairs) are left blank. Color intensity follows a diverging scale from blue (lower CSE) through white to red (higher CSE), as indicated by the shared color bar on the left. Bold black borders highlight region pairs exhibiting significant post hoc differences between the stable MDD and diagnosed BD groups after Holm–Bonferroni correction: RF–RP (corrected *p* = 0.049) and RP–RC (corrected *p* = 0.047). Asterisks (*) in the diagnosed BD panel mark these significant pairs. All omnibus group comparisons were performed using analysis of covariance with mean-centered Beck Depression Inventory score as a covariate. Inferential *p*-values were obtained via a restricted-residual wild bootstrap procedure (20 000 replicates with Mammen 2-point distribution weights), and post hoc pairwise contrasts were corrected using the Holm–Bonferroni method. Figure 2. long description.

Intra-regional CSE showed significant omnibus effects in 5 of 6 regions; only the left central region was non-significant (*p* = 0.110). Covariate-adjusted CSE values were lower in diagnosed BD than in stable MDD across significant regions, with converted BD again intermediate. After Holm–Bonferroni correction, only the right frontal region remained significant for diagnosed BD versus stable MDD (*F*(2, 88) = 5.98, *η*^2^ₚ = 0.120, *p* = 0.023; *p*_Holm = 0.049) (Figure 2A). No other corrected pairwise contrasts were significant.

Inter-regional CSE showed significant omnibus effects in 11 of 15 region pairs; all 4 non-significant pairs involved the left central region. As in the intra-regional analysis, covariate-adjusted values were lower in diagnosed BD than in stable MDD, with converted BD intermediate. Corrected post hoc differences between diagnosed BD and stable MDD were identified for right parietal–right central (*F*(2, 88) = 5.23, *η*^2^ₚ = 0.106, *p* = 0.022; *p*_Holm = 0.047) and right frontal–right parietal (*F*(2, 88) = 5.84, *η*^2^ₚ = 0.117, *p* = 0.022; *p*_Holm = 0.049) (Figure 2B). No other corrected pairwise contrasts were significant.

### Sensitivity of entropy estimates to segment selection

Robustness to segment selection was assessed by repeated random subsampling of artifact-free 30-second segments. For each *k* from 1 to 8, *k* segments were drawn without replacement from each participant across 100 iterations, and subject-level entropy values were defined as the median across sampled segments. The within-subject coefficient of variation (CV) across iterations was calculated for each entropy feature to quantify segment-selection variability. Analyses were limited to participants with at least *k* retained segments, with at least 71 participants contributing at every *k.*

Segment-selection variability was low and decreased monotonically as *k* increased. Mean within-subject CV across all 45 entropy features declined from 2.21% to 0.39% for band-scale entropy and from 4.32% to 0.86% for CSE between *k* = 1 and *k* = 8. By *k* = 3, mean CVs had already fallen to 1.25% and 2.58%, respectively. Band-scale entropy showed lower variability than CSE at all *k* values, and variability was comparable across diagnostic groups.

Convergence with full-data estimates was high even at small *k.* The mean correlation between subsampled and full-data subject-level entropy estimates was *r* = 0.921 at *k* = 1, *r* = 0.968 at *k* = 3, *r* > 0.98 at *k* = 5, and *r* = 0.995 at *k* = 8.

As an unadjusted sensitivity analysis, stability of group separation between stable MDD and diagnosed BD was assessed by repeating Mann–Whitney *U* tests at each *k.* For the 4 band-scale entropy features that showed significant covariate-adjusted group differences in the primary analysis, unadjusted significance (*p* < 0.05) was reproduced in 100% of iterations at every tested *k.* For the 3 significant CSE features (intra-regional right frontal, inter-regional right frontal–right parietal, and inter-regional right parietal–right central), replication rates ranged from 46% to 73% at *k* = 1, increased to 74–97% by *k* = 4, and exceeded 80% for 2 of the 3 features at *k* = 6. These results indicated greater segment-selection sensitivity for CSE than for band-scale entropy.

### Associations between entropy measures and psychological assessments

**Figure 3.**
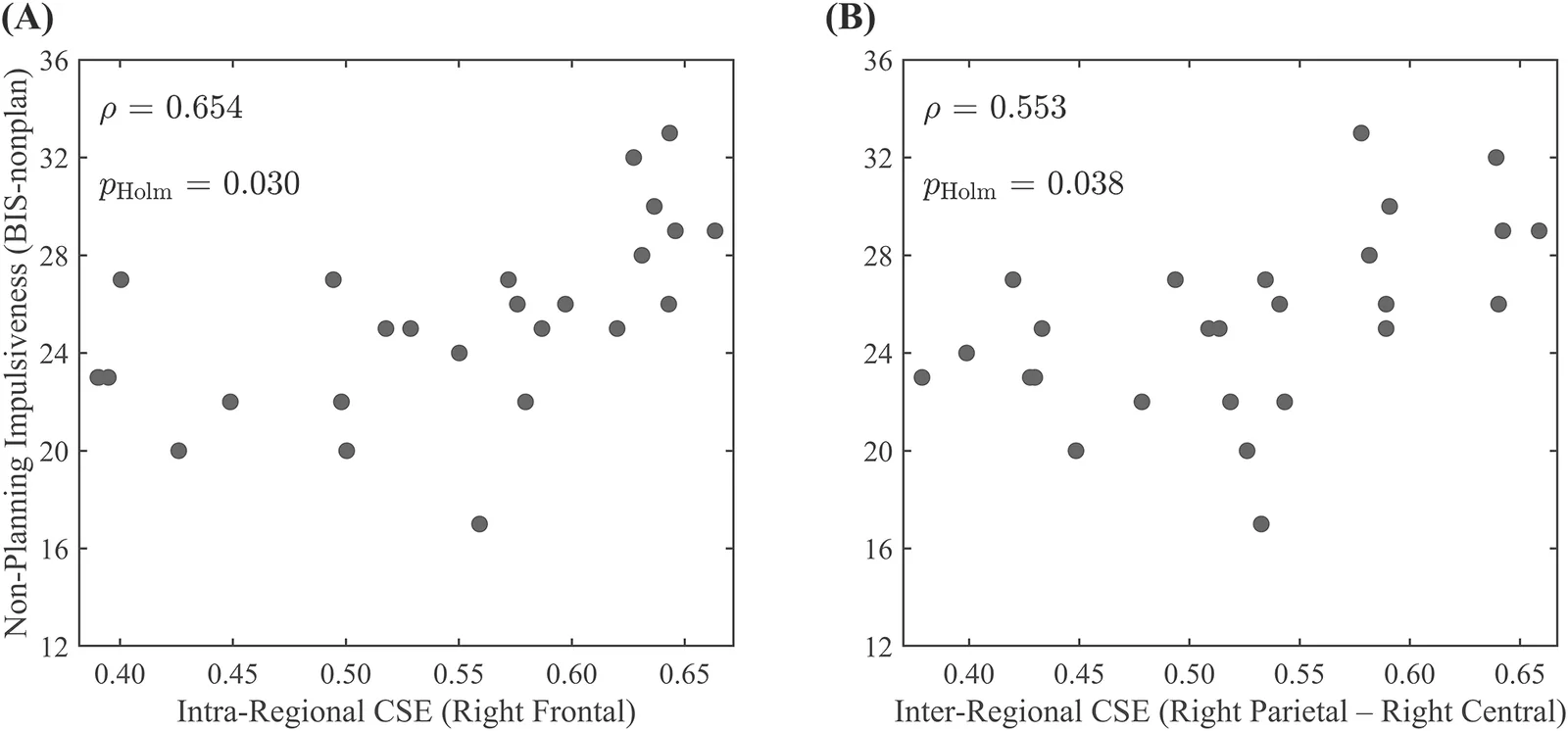
Positive associations between cross-sample entropy measures and Non-Planning Impulsiveness in the diagnosed bipolar disorder group. (A) Scatter plot depicting the correlation between intra-regional cross-sample entropy (CSE) in the right frontal region and Non-Planning Impulsiveness scores from the Barratt Impulsiveness Scale, version 11 (BIS-11) (Spearman *ρ* = 0.654, *p*_Holm = 0.030). (B) Scatter plot depicting the correlation between inter-regional CSE of the right parietal–right central connection and Non-Planning Impulsiveness scores (Spearman *ρ* = 0.553, *p*_Holm = 0.038). Each data point represents an individual participant with diagnosed bipolar disorder (*n* = 26). Spearman rank correlation coefficients (*ρ*) and *p*-values corrected for multiple comparisons across 7 clinical scales using the Holm–Bonferroni method (*p*_Holm) are displayed in each panel. Inferential *p*-values were derived from a restricted-residual wild bootstrap procedure applied to rank-transformed variables (20 000 replicates with Mammen 2-point distribution weights). Figure 3. long description.

Correlation analyses were restricted to the 7 entropy features that showed Holm–Bonferroni-corrected post hoc differences between diagnosed BD and stable MDD in section “Entropy-based EEG measures for mood disorder differentiation”. Each feature was correlated with 7 clinical measures—state anxiety (STAI-S), trait anxiety (STAI-T), BDI, BIS-attentional, BIS-motor, BIS-nonplan, and BIS-11 total score—separately within the 2 groups involved in the significant post hoc contrasts (stable MDD and diagnosed BD). In the stable MDD group, neither the global test nor any pairwise association was significant. In the diagnosed BD group (*n* = 26), the global test across all 49 feature–clinical-measure pairs was significant (sum-*T*^2^ = 141.10, *p* = 0.031; 20 000 wild bootstrap replicates with Mammen weights). After Holm–Bonferroni correction across the 7 clinical scales within each feature, 2 positive associations remained significant, both involving BIS-nonplan. Intra-regional CSE in the right frontal region was positively correlated with BIS-nonplan (*ρ* = 0.654, *p* = 0.004, *p*_Holm = 0.030) (Figure 3A), and inter-regional CSE between the right parietal and right central regions showed a similar positive association (*ρ* = 0.553, *p* = 0.005, *p*_Holm = 0.038) (Figure 3B). No band-scale entropy feature was significantly associated with any clinical measure after correction, and no other corrected CSE association was significant.

### Machine-learning classification of mood disorder groups

**Figure 4.**
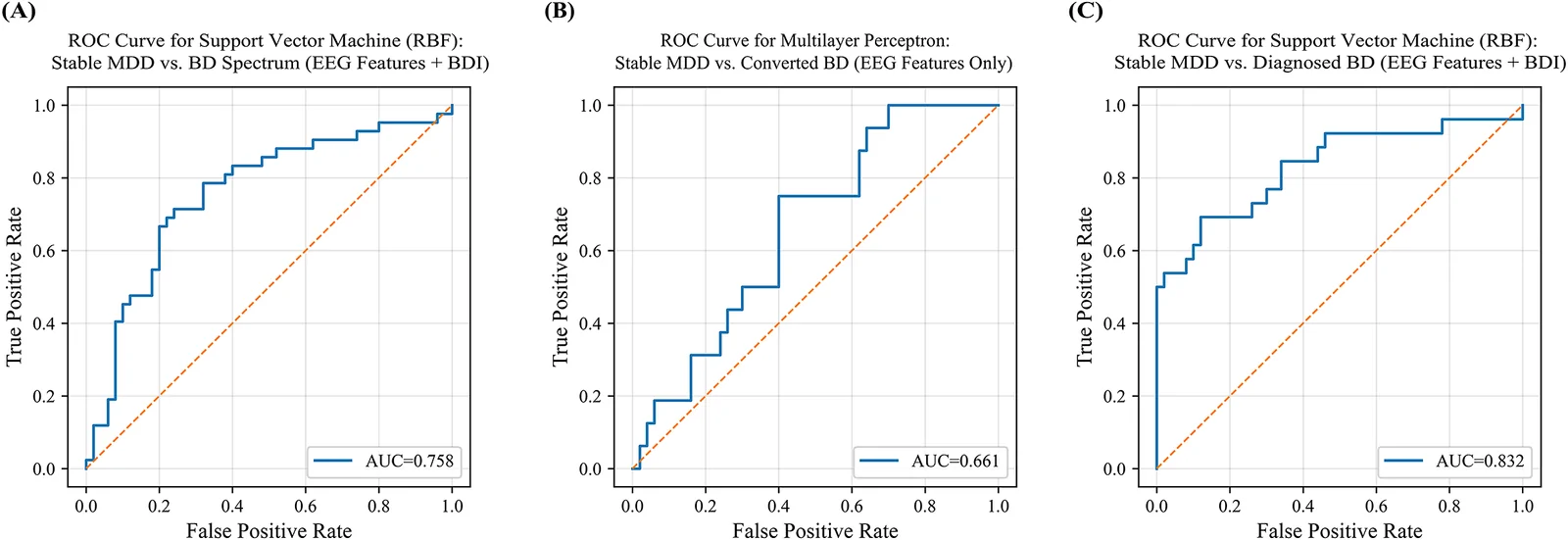
Receiver operating characteristic (ROC) curves for machine learning classification of mood disorder groups using entropy-derived EEG features, with Beck Depression Inventory (BDI) score included as an additional predictor in panels (A) and (C). (A) Stable major depressive disorder (stable MDD; *n* = 50) versus bipolar disorder (BD) spectrum (converted BD and diagnosed BD combined; *n* = 42), classified by a support vector machine with a radial basis function kernel (RBF-SVM) using EEG features + BDI (AUC = 0.758; total *n* = 92). (B) Stable MDD (*n* = 50) versus converted BD (*n* = 16), classified by a multilayer perceptron (MLP) using the EEG-only configuration (AUC = 0.661; total *n* = 66). (C) Stable MDD (*n* = 50) versus diagnosed BD (*n* = 26), classified by RBF-SVM using EEG features + BDI (AUC = 0.832; total *n* = 76). In each panel, the blue solid line represents the ROC curve derived from pooled subject-level out-of-fold decision scores, and the orange dashed diagonal indicates chance performance (AUC = 0.5). Models were evaluated using repeated stratified nested cross-validation (5 folds × 20 repeats). Permutation testing based on balanced accuracy yielded *p* = 0.005, 0.040, and 0.005 for panels (A), (B), and (C), respectively. Figure 4. long description.

Across nested cross-validation (5 folds × 20 repeats), the optimal pipeline retained the complete feature set in all 3 classification tasks; the full classifier-family comparison is reported in Supplementary Table S1, and the corresponding feature-selection and resampling comparisons are reported in Supplementary Tables S2 and S3, respectively. For stable MDD versus BD spectrum (*n* = 92), the best model was an RBF-SVM using EEG + BDI and no resampling. Pooled balanced accuracy was 71.5% (95% CI: 61.8–81.3%; mean = 66.6 ± 10.8%) and pooled ROC-AUC was 0.758 (95% CI: 0.648–0.861; mean = 0.709 ± 0.152), with sensitivity of 69.0% (95% CI: 54.3–83.3%) and specificity of 74.0% (95% CI: 60.4–85.7%). Overall pooled accuracy was 71.7% (95% CI: 62.0–81.5%; mean = 67.1 ± 10.8%) and pooled F1-score was 69.0% (95% CI: 56.7–80.0%; mean = 63.5 ± 12.7%). Permutation testing confirmed above-chance balanced accuracy (*p* = 0.005) (Figure 4A). The EEG + BDI configuration outperformed the EEG-only configuration (pooled balanced accuracy = 69.7%; ROC-AUC = 0.667) and the BDI-only configuration (pooled balanced accuracy = 60.2%; ROC-AUC = 0.619).

For stable MDD versus converted BD (*n* = 66), the best model was an MLP using the EEG-only configuration with Borderline-SMOTE. BDI was excluded from this task because both groups presented with comparable depressive symptom severity at the time of recording, limiting its expected discriminative value (see section “Feature selection and classification”). Pooled balanced accuracy was 65.4% (95% CI: 52.7–79.1%; mean = 62.6 ± 13.6%) and pooled ROC-AUC was 0.661 (95% CI: 0.519–0.798; mean = 0.669 ± 0.154), with sensitivity of 68.8% (95% CI: 46.1–91.7%) and specificity of 62.0% (95% CI: 47.9–75.0%). Overall pooled accuracy was 63.6% (95% CI: 53.0–75.8%; mean = 61.6 ± 13.6%) and pooled F1-score was 47.8% (95% CI: 28.6–64.2%; mean = 46.2 ± 14.9%). Permutation testing indicated above-chance balanced accuracy (*p* = 0.040) (Figure 4B).

For stable MDD versus diagnosed BD (*n* = 76), the best model was an RBF-SVM using EEG + BDI with SMOTE. Pooled balanced accuracy was 76.6% (95% CI: 65.9–87.2%; mean = 71.3 ± 13.5%) and pooled ROC-AUC was 0.832 (95% CI: 0.709–0.930; mean = 0.769 ± 0.181), with sensitivity of 69.2% (95% CI: 50.0–87.5%) and specificity of 84.0% (95% CI: 72.9–93.8%). Overall pooled accuracy was 78.9% (95% CI: 69.7–88.2%; mean = 72.5 ± 15.6%) and pooled F1-score was 69.2% (95% CI: 52.6–82.4%; mean = 63.7 ± 15.7%). Permutation testing confirmed above-chance balanced accuracy (*p* = 0.005) (Figure 4C). The EEG + BDI configuration outperformed the EEG-only configuration (pooled balanced accuracy = 59.3%; ROC-AUC = 0.670) and the BDI-only configuration (pooled balanced accuracy = 59.7%; ROC-AUC = 0.698).

In ablation analyses, the combined feature set outperformed single-family configurations in all 3 tasks. For the stable MDD versus BD spectrum and stable MDD versus diagnosed BD tasks, BDI score was retained in every ablation configuration so that performance differences would reflect the contribution of EEG feature families rather than the presence or absence of BDI; the stable MDD versus converted BD task used EEG features only, as described in section “Feature selection and classification.” For stable MDD versus BD spectrum, the band-scale entropy + BDI configuration yielded pooled balanced accuracy of 66.7% (95% CI: 56.5–76.4%; mean = 58.9 ± 10.8%) and pooled ROC-AUC of 0.692 (95% CI: 0.574–0.807; mean = 0.573 ± 0.192), the CSE + BDI configuration yielded 68.0% (95% CI: 57.7–77.0%; mean = 63.6 ± 10.2%) and 0.721 (95% CI: 0.610–0.819; mean = 0.710 ± 0.136), and the combined entropy + BDI configuration reached 71.5% and 0.758. For stable MDD versus diagnosed BD, the band-scale entropy + BDI configuration yielded 67.8% (95% CI: 56.3–79.1%; mean = 65.5 ± 11.5%) and 0.775 (95% CI: 0.646–0.890; mean = 0.698 ± 0.202), the CSE + BDI configuration yielded 68.8% (95% CI: 57.4–79.2%; mean = 70.4 ± 11.7%) and 0.822 (95% CI: 0.715–0.909; mean = 0.820 ± 0.102), and the combined entropy + BDI configuration reached 76.6% and 0.832. For stable MDD versus converted BD, the single-family models showed lower, near-chance performance (band-scale entropy: 41.8%, 95% CI: 28.2–55.2%, mean = 45.7 ± 13.2%; ROC-AUC = 0.453, 95% CI: 0.286–0.611, mean = 0.459 ± 0.185; CSE: 52.0%, 95% CI: 38.0–66.5%, mean = 53.1 ± 14.0%; ROC-AUC = 0.573, 95% CI: 0.413–0.734, mean = 0.570 ± 0.170), whereas the combined feature set performed better (balanced accuracy 65.4%, ROC-AUC 0.661).

**Figure 5.**
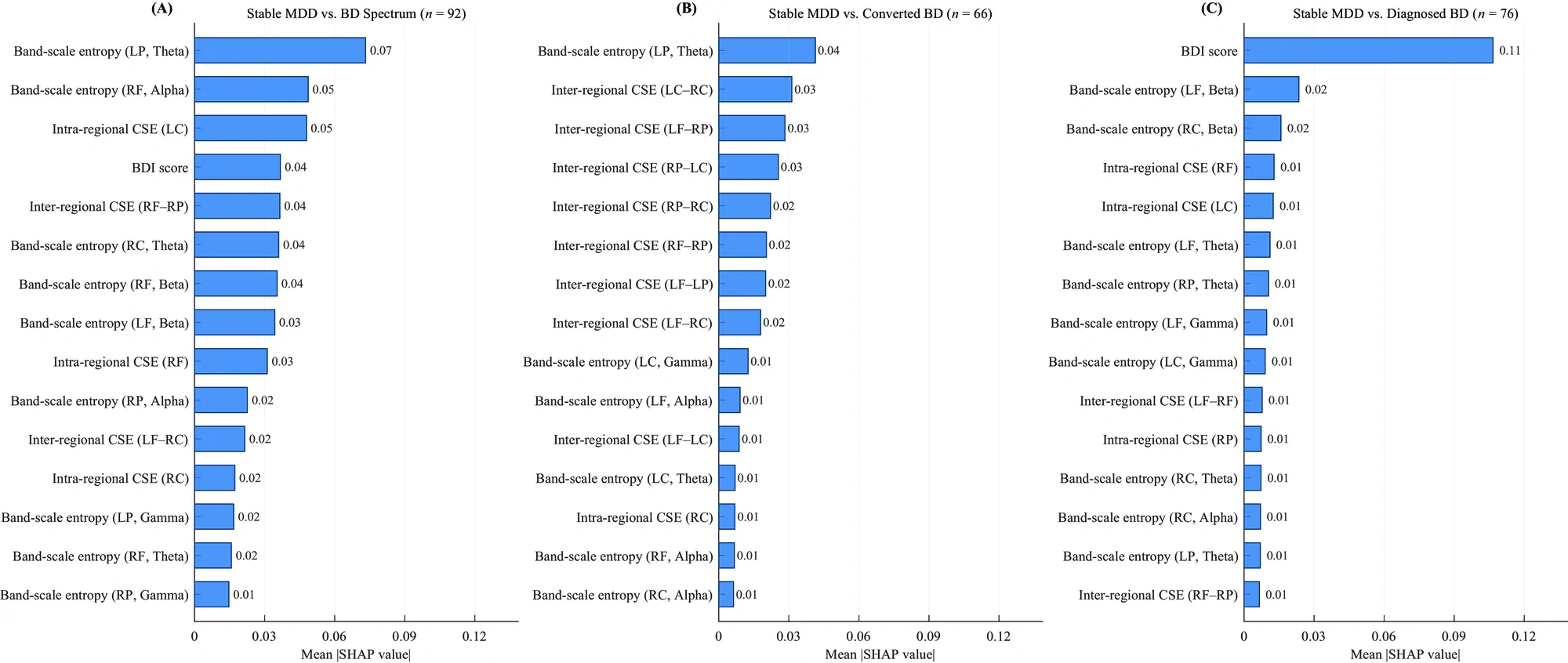
Mean absolute SHAP feature importance for the best-performing classifiers across 3 binary classification tasks distinguishing mood disorder groups. Each panel displays the top 15 features ranked by mean absolute Shapley Additive Explanations (SHAP) value, quantifying the average magnitude of the contribution of each feature to model output. (A) Stable major depressive disorder (stable MDD) versus bipolar disorder (BD) spectrum (combined converted BD and diagnosed BD; *n* = 92), classified by a support vector machine with a radial basis function kernel (RBF-SVM). (B) Stable MDD versus converted BD (*n* = 66), classified by a multilayer perceptron (MLP). (C) Stable MDD versus diagnosed BD (*n* = 76), classified by RBF-SVM. Horizontal bars represent the mean |SHAP value| for each feature, with numerical values annotated at bar ends. The x-axis scale is shared across all 3 panels to facilitate direct comparison of feature importance magnitudes across tasks. The predictor set comprised 45 entropy-derived EEG features; the Beck Depression Inventory (BDI) score was included as an additional predictor in panels (A) and (C) and excluded from panel (B). Feature labels denote band-scale entropy (brain region, frequency band) or cross-sample entropy (CSE); intra-regional CSE refers to mean pairwise CSE within a region, and inter-regional CSE refers to mean pairwise CSE between regions. Region abbreviations: LF, left frontal; RF, right frontal; LC, left central; RC, right central; LP, left parietal; RP, right parietal. Figure 5. long description.

SHAP analysis indicated task-specific but complementary contributions from band-scale entropy and CSE. For stable MDD versus BD spectrum, left parietal theta-band entropy showed the largest mean absolute SHAP value (0.073), followed by right frontal alpha-band entropy (0.049) and intra-regional left central CSE (0.048); BDI ranked fourth (0.037) (Figure 5A). For stable MDD versus converted BD, left parietal theta-band entropy again ranked first (0.041), followed by left central–right central CSE (0.031) and left frontal–right parietal CSE (0.028) (Figure 5B). For stable MDD versus diagnosed BD, BDI ranked first (0.107), followed by left frontal beta-band entropy (0.024), right central beta-band entropy (0.016), and intra-regional right frontal CSE (0.013) (Figure 5C). Band-scale entropy predominated among the top 10 EEG contributors in the first and third tasks (6/10 each), whereas CSE predominated in the second task (7/10). The highest-ranked classifier features only partially overlapped with the 7 entropy features showing corrected univariate group differences in section “Entropy-based EEG measures for mood disorder differentiation.”

Robustness analyses showed stable classification for the first and third tasks and greater variability for the second. Under 100 random segment-sampling iterations, mean pooled balanced accuracy and ROC-AUC were 66.3% (95% CI: 56.5–75.8%; mean = 61.3 ± 11.2%) and 0.697 (95% CI: 0.582–0.805; mean = 0.630 ± 0.173) for stable MDD versus BD spectrum, with sensitivity of 64.1% (95% CI: 49.3–78.2%) and specificity of 68.6% (95% CI: 55.4–81.0%); 57.8% (95% CI: 44.2–71.0%; mean = 56.0 ± 14.1%) and 0.590 (95% CI: 0.436–0.738; mean = 0.593 ± 0.172) for stable MDD versus converted BD, with sensitivity of 62.7% (95% CI: 39.0–85.1%) and specificity of 52.9% (95% CI: 39.2–66.4%); and 73.5% (95% CI: 62.6–83.7%; mean = 69.3 ± 12.0%) and 0.808 (95% CI: 0.689–0.911; mean = 0.756 ± 0.166) for stable MDD versus diagnosed BD, with sensitivity of 68.5% (95% CI: 49.8–85.7%) and specificity of 78.5% (95% CI: 66.9–89.1%). Across 10 random seeds, the mean pooled balanced accuracy across seeds was 71.2 ± 1.6%, 54.6 ± 5.8%, and 72.1 ± 2.0% for the 3 tasks, respectively; the corresponding fold-wise mean balanced accuracies were 65.7 ± 1.0%, 53.4 ± 2.9%, and 69.7 ± 1.2%. The mean pooled ROC-AUC across seeds was 0.757 ± 0.011, 0.573 ± 0.055, and 0.814 ± 0.006, respectively; the corresponding fold-wise mean ROC-AUC values were 0.703 ± 0.012, 0.562 ± 0.040, and 0.770 ± 0.014. Cross-seed sensitivity and specificity remained stable for the first task (69.3 ± 4.0% and 73.2 ± 2.5%) and third task (66.5 ± 4.8% and 77.6 ± 3.1%), whereas the second task exhibited greater variability (50.0 ± 12.8% and 59.2 ± 7.2%), consistent with its smaller sample size and stronger class imbalance.

## Discussion

The present study used entropy-based EEG analysis to investigate neurophysiological differences among drug-naïve patients with stable MDD, converted BD, and diagnosed BD. A global set-level test across all 45 entropy features revealed a significant omnibus group effect after adjustment for depressive symptom severity (sum-*F* = 173.73, *p* = 0.026; 20 000 wild bootstrap replicates). Band-scale entropy was significantly elevated in the diagnosed BD group relative to the stable MDD group, predominantly in the theta and alpha bands, whereas no significant effects emerged in the beta or gamma bands. Conversely, selected intra- and inter-regional CSE measures were significantly lower in the diagnosed BD group, indicating more regular and predictable neural dynamics. Within the diagnosed BD group, CSE measures correlated positively with non-planning impulsiveness. The converted BD group consistently exhibited covariate-adjusted values intermediate between the other 2 groups; however, no pairwise contrast involving this group reached statistical significance. Machine learning classification was strongest for patients with stable MDD versus those with diagnosed BD (pooled accuracy 78.9%, balanced accuracy 76.6%, ROC-AUC 0.832). Collectively, these entropy-derived patterns support a biologically meaningful distinction between unipolar and bipolar depression detectable in a drug-naïve depressive-state cohort.

The regional pattern of band-scale entropy suggests that bipolar depression is characterized primarily by altered theta- and alpha-range complexity rather than by a diffuse increase across all frequency bands. Bilateral frontal theta elevation in diagnosed BD aligns with prior evidence of frontal theta involvement in reward evaluation, cognitive control, and affective regulation—domains repeatedly implicated in bipolar depression.^76^^,^^77^ Abnormal theta power modulation in BD has been associated with impaired reward processing and heightened negative-feedback sensitivity during depressive episodes, and deficient cognitive inhibition during euthymia.^76^^,^^78^ Importantly, these effects remained significant after covariate adjustment for depressive symptom severity, suggesting that theta-band entropy elevation is not fully explained by measured depressive severity, although residual symptom-severity confounding cannot be excluded. The concomitant increase in alpha-band entropy is interpretable in light of the established positive correlation between band-scale entropy and spectral power within corresponding frequency bands (*r* = 0.709 for alpha, *p* < 0.001), which indicates that elevated alpha-band entropy likely reflects increased alpha-range oscillatory power.^12^ This interpretation aligns with reports of higher alpha power in BD relative to MDD across widespread cortical regions;^17^ given the established role of alpha oscillations in cortical inhibition and the timing of attentional selection,^79^ this elevated alpha-range activity may index altered inhibitory gating of sensory and cognitive processing in BD.

The CSE findings provide a complementary interpretation of inter-electrode interaction dynamics. Lower CSE in diagnosed BD was concentrated in right frontal intra-regional dynamics and in right frontoparietal and parietal–central interactions, indicating more regular cross-signal dynamics consistent with reduced flexibility of right-lateralized communication. This right-lateralized pattern is consistent with the proposed involvement of the right hemisphere in emotional processing and arousal modulation and with evidence that atypical right-hemispheric functioning constitutes a neurobehavioral vulnerability marker of emotional dysregulation in BD.^80^^,^^81^ The observation of lower CSE values in the diagnosed BD group aligns with evidence that periods of emotional distress are characterized by reduced CSE,^20^ extending the previously reported enhancements in interhemispheric coordination to the right-lateralized networks observed here. The pattern also converges with FC evidence of elevated interamygdala coupling in populations at risk for BD and of default mode–frontoparietal network hyperconnectivity in euthymic patients with BD; the latter has been proposed as a trait marker.^14^^,^^82^ Because CSE displayed greater segment-selection sensitivity than band-scale entropy, this interpretation should be regarded as biologically plausible but methodologically more provisional.

Within the diagnosed BD group, CSE correlated positively with BIS-nonplan (right frontal intra-regional: *ρ* = 0.654, *p*_Holm = 0.030; right parietal–right central inter-regional: *ρ* = 0.553, *p*_Holm = 0.038), whereas no association remained statistically significant after correction in the stable MDD group or for any band-scale entropy feature. The positive correlation may appear to conflict with the overall lower CSE in diagnosed BD; however, between-group differences indicate a downward shift of the synchrony regime, while within-group correlations capture individual variability whereby relatively less synchronized patients exhibit greater impulsivity. This dissociation supports a nonlinear relation between neural synchrony and behavioral regulation in bipolar depression, consistent with evidence that neural synchronization may have an optimal range, with deviations in either direction contributing to different aspects of pathology.^83^ The absence of significant band-scale entropy–clinical associations after correction indicates that CSE may be more directly related to behavioral manifestations than single-channel complexity metrics, underscoring the complementary roles of the 2 entropy measures: band-scale entropy captures intrinsic signal complexity differences between diagnostic groups, whereas CSE bridges neural dynamics and observable clinical behavior.

The converted BD group consistently exhibited intermediate entropy values, although pairwise contrasts involving this group did not reach statistical significance, likely reflecting limited statistical power (*n* = 16). The classifier for patients with stable MDD versus those with converted BD also demonstrated the lowest performance (balanced accuracy 65.4%, ROC-AUC 0.661), and across seeds it showed the lowest mean pooled balanced accuracy (54.6%) together with the greatest variability (SD = 5.8%). This ordinal pattern is descriptively consistent with the hypothesis that the transition from MDD to BD represents a neurobiological continuum; however, these intermediate values remained statistically unconfirmed in pairwise comparisons. Particularly in the theta band, this lack of statistical significance may suggest that theta-related neural complexity changes become manifest only after the bipolar phenotype has fully emerged. These findings indicate that entropy-based biomarkers capture a weak but detectable conversion-related signal requiring larger prospective cohorts for definitive evaluation.

The optimal pipeline retained the complete feature set in all 3 tasks, indicating that discriminatory information was distributed across many weak-to-moderate predictors. Combined use of band-scale entropy and CSE consistently outperformed either single-family configuration across all 3 tasks. This was most notable for the stable MDD versus converted BD task, where the single-family models showed lower, near-chance performance, whereas the combined feature set yielded comparatively better classification. Furthermore, the EEG + BDI configuration outperformed both EEG-only and BDI-only configurations whenever depressive severity differed across groups. The partial overlap between top SHAP features and corrected univariate group differences further indicates that multivariate classification relied on combinatorial information not fully captured by single-feature inference.

Direct comparison with prior differentiation studies places the current performance within a competitive range. An EEG coherence-based study using nested cross-validation achieved 80.19% accuracy and an AUC of 0.793 in distinguishing 46 patients with BD from 55 with MDD.^18^ An fMRI-based SVM classifier trained on amygdala FC achieved 73.5% mean accuracy (AUC 0.735) across 48 BD and 50 unipolar depression patients, reaching 81% accuracy on an independent conversion sample.^15^ More recently, a comprehensive resting-state fMRI study using 5-fold nested cross-validation with permutation testing found that only seed-based connectivity reached significance (balanced accuracy 66.22%, AUC 0.71),^16^ noting that prior smaller-sample results could be inflated. In this context, the current pooled accuracy of 78.9%, balanced accuracy of 76.6%, and AUC of 0.832 appear competitive, although external validation is still required to determine how well these estimates generalize to independent cohorts. Direct numerical comparison should be interpreted cautiously given differences in imaging modality, sample composition, medication status, and validation rigor. The current analysis incorporated repeated nested cross-validation, training-fold threshold optimization, permutation testing, and multi-faceted robustness analyses, which collectively minimize data leakage and overfitting risk.

A major novelty lies in the simultaneous inclusion of stable MDD, converted BD, and diagnosed BD under drug-naïve conditions, combined with multilevel quantification of local signal complexity and inter-regional interaction complexity. The drug-naïve design eliminates medication-related confounds that can influence EEG patterns and inflate or distort classifier performance in medicated cohorts. Additional methodological strength arises from diagnosis-blind automated preprocessing and from the integration of global testing, bootstrap-based inference, repeated nested cross-validation, ablation analysis, SHAP-based interpretability assessment, and robustness checks within a single analytic framework. In clinical terms, the most defensible interpretation is that entropy-derived EEG markers may function as adjunctive biomarkers that complement, rather than replace, longitudinal symptom assessment and course-based diagnosis.

### Limitations

Several limitations should temper the interpretation of these findings. The converted BD sample was small and imbalanced relative to the other groups, likely reducing power for pairwise contrasts and contributing to instability in the conversion-focused classifier. Phenotypic heterogeneity remained because converted BD comprised only BD-II, whereas diagnosed BD included both BD-I and BD-II. Diagnostic conversion was confirmed under DSM-5 criteria that allow BD-II diagnosis after antidepressant-emergent hypomania, enhancing clinical realism but leaving open whether all converted cases map onto the same biological phenotype as spontaneous bipolarity. Because no external validation cohort was available, repeated nested cross-validation across multiple random seeds (seeds 0–9) and segment-resampling analyses were used to probe internal reproducibility (e.g., pooled ROC-AUC = 0.757 ± 0.011 for stable MDD versus BD spectrum; 0.814 ± 0.006 for stable MDD versus diagnosed BD); however, these procedures do not eliminate possible site- or recording-dependence, and cross-site validation would have been preferable. Because depressive severity differed between diagnosed BD and stable MDD, residual concern about symptom-severity confounding remains, although the EEG + BDI configuration outperformed both EEG-only and BDI-only configurations, indicating that classification performance cannot be reduced to depressive severity alone. Finally, greater segment-selection sensitivity for CSE than for band-scale entropy indicates that inter-electrode complexity markers may require especially careful stabilization in future protocols.

## Conclusion and future work

Entropy-based EEG analysis revealed distinct neurophysiological signatures across mood disorder subtypes in drug-naïve patients, with the diagnosed BD group characterized by elevated theta- and alpha-band complexity and decreased CSE, whereas the converted BD group showed descriptively intermediate values that did not reach statistical significance. Machine learning classification achieved above-chance balanced accuracy in the primary nested cross-validation analyses, but discrimination of stable MDD versus converted BD was modest and less stable in robustness analyses; combined entropy features outperformed either single-family entropy configuration. These findings highlight the potential of entropy-based measures as objective, non-invasive biomarkers for mood disorder differentiation.

Future research should prioritize multi-site data collection for cross-site validation, the establishment of large-scale prospective cohorts targeting the pre-conversion window, longitudinal tracking of entropy measures, and multimodal biomarker integration of EEG with neuroimaging and clinical phenotyping. Extension to multi-class classification and task-based EEG paradigms would bring the framework closer to real-world diagnostic decision-making and support translation of entropy-based neural signatures into clinically actionable decision-support tools for the early identification of bipolar conversion risk.

## Supporting information

10.1017/S1092852926100923.sm001 Hwang et al. supplementary material Hwang et al. supplementary material

## Data Availability

To ensure the privacy and ethical obligations of the participants, the datasets from this study were not publicly accessible and were securely stored in the Brain Signals and Mental Health Laboratory database at Soonchunhyang University Cheonan Hospital. Researchers interested in accessing de-identified versions of these datasets for scientific purposes may submit formal requests to Ji Sun Kim, MD, PhD, at ideal91@hanmail.net.

## Supplementary material

The supplementary material for this article can be found at http://doi.org/10.1017/S1092852926100923.

## Long description

### Table 1. Long description

**Table:** Table 1 compares demographic, clinical, and EEG recording characteristics across the stable MDD, converted BD, and diagnosed BD groups. Continuous variables are presented as mean ± standard deviation, and sex is presented as male/female counts.

Variable	Stable MDD(n = 50)	Converted BD(n = 16)	Diagnosed BD(n = 26)	P	Post hoc
Sex (male/female)	14/36	5/11	11/15	0.447	—
Age (years)	27.64 ± 6.99	24.44 ± 4.72	31.15 ± 10.41	0.101	—
Education (years)	13.50 ± 2.26	13.25 ± 1.77	13.62 ± 1.96	0.749	—
State anxiety (STAI-S)	63.38 ± 9.96	61.19 ± 7.83	58.92 ± 11.00	0.180	—
Trait anxiety (STAI-T)	63.64 ± 8.78	63.94 ± 8.19	58.58 ± 11.42	0.145	—
**BDI**	**49.60 ± 10.53**	**48.50 ± 10.53**	**35.27 ± 16.45**	**0.001**	**Stable MDD > diagnosed BD; converted BD > diagnosed BD**
BIS-motor	29.56 ± 5.71	28.81 ± 3.76	29.15 ± 3.28	0.942	—
BIS-nonplan	25.68 ± 4.82	23.88 ± 3.61	25.23 ± 3.72	0.355	—
BIS-attentional	20.94 ± 4.39	21.00 ± 3.81	20.81 ± 3.06	0.810	—
BIS–11 total score	76.18 ± 12.49	73.69 ± 8.84	75.19 ± 7.89	0.842	—
No. of artifact-free 30-s segments	8.40 ± 2.55	8.62 ± 2.16	9.00 ± 2.87	0.176	—

**Note.** P-values are from the chi-square test, one-way ANOVA, Welch’s ANOVA, or Kruskal-Wallis test, selected according to data type and distributional properties. Significant omnibus tests were followed by pairwise Welch t-tests.

**Note.** MDD, major depressive disorder; BD, bipolar disorder; BD-I, bipolar I disorder; BD-II, bipolar II disorder; STAI, State-Trait Anxiety Inventory; BDI, Beck Depression Inventory; BIS-11, Barratt Impulsiveness Scale, version 11; No., number. All participants in converted BD had BD-II. Diagnosed BD included 7 participants with BD-I and 19 with BD-II.

Navigate back to Table 1..

### Figure 1. Long description

Figure 1 contains six grouped box-plot panels in a 2 × 3 grid: left frontal, central, and parietal regions on the top row and the corresponding right-sided regions on the bottom. Each panel compares theta, alpha, beta, and gamma band-scale entropy in stable MDD (blue; n = 50), converted BD (red; n = 16), and diagnosed BD (green; n = 26). Boxes show interquartile ranges and medians; whiskers and jittered points show dispersion and individual participants. Holm-Bonferroni-corrected brackets indicate higher diagnosed BD than stable MDD for left frontal theta (p = 0.030), left frontal alpha (p = 0.031), right frontal theta (p = 0.031), and right central alpha (p = 0.037). Converted BD is generally intermediate, but no corrected contrast involving converted BD is significant. No other corrected pairwise difference is marked.

Navigate back to Figure 1..

### Figure 2. Long description

Figure 2 compares cross-sample entropy (CSE) in stable MDD (blue; n = 50), converted BD (red; n = 16), and diagnosed BD (green; n = 26). Panel A shows violin plots of intra-regional CSE for left and right frontal, central, and parietal regions, with points for individuals, white median circles, interquartile bars, and whiskers. A bracket marks lower right frontal CSE in diagnosed BD than stable MDD (Holm-Bonferroni-corrected p = 0.049). Panel B shows group-mean inter-regional CSE heatmaps for six regions: LF, RF, LC, RC, LP, and RP. Blue indicates lower and red higher CSE; diagonal cells are blank. In diagnosed BD, bordered cells and asterisks mark lower RF-RP CSE (p = 0.049) and RP-RC CSE (p = 0.047) than stable MDD. Converted BD is generally intermediate, with no corrected contrast involving that group.

Navigate back to Figure 2..

### Figure 3. Long description

Figure 3 contains two scatter plots for participants with diagnosed bipolar disorder (BD; n = 26). Each point represents one participant, and both panels show positive associations with the Non-Planning Impulsiveness subscale of the Barratt Impulsiveness Scale, version 11. Panel A plots right frontal intra-regional cross-sample entropy (CSE) against Non-Planning Impulsiveness; Spearman rho = 0.654 and the Holm-Bonferroni-corrected p value is 0.030. Panel B plots right parietal-right central inter-regional CSE against Non-Planning Impulsiveness; Spearman rho = 0.553 and the corrected p value is 0.038. Thus, participants with higher values of either CSE measure tend to have higher non-planning impulsiveness scores. Inferential p values were obtained by a restricted-residual wild bootstrap applied to rank-transformed variables using 20,000 replicates.

Navigate back to Figure 3..

### Figure 4. Long description

Figure 4 contains three receiver operating characteristic (ROC) curves. True positive rate is plotted against false positive rate; the blue line is the pooled out-of-fold ROC curve and the orange diagonal is chance performance (AUC = 0.5). Panel A compares stable MDD (n = 50) with the BD spectrum (n = 42) using an RBF-SVM with EEG features plus BDI: AUC = 0.758 and permutation p = 0.005. Panel B compares stable MDD (n = 50) with converted BD (n = 16) using an MLP with EEG-only features: AUC = 0.661 and p = 0.040. Panel C compares stable MDD (n = 50) with diagnosed BD (n = 26) using an RBF-SVM with EEG features plus BDI: AUC = 0.832 and p = 0.005. Models were evaluated by repeated stratified nested cross-validation (5 folds × 20 repeats).

Navigate back to Figure 4..

### Figure 5. Long description

Figure 5 contains three horizontal bar charts showing the 15 highest-ranked predictors for the best-performing classifiers. Bar length represents mean absolute SHAP value, which shows the average magnitude, not the direction, of each feature’s contribution. The shared x-axis ranges from 0 to 0.12; values printed at the bar ends are reproduced below.

**Table:**

Panel A: Stable MDD vs. BD spectrum (n = 92), RBF-SVM		
Rank	Feature	Mean |SHAP value|
**1**	**Band-scale entropy (LP, Theta)**	**0.07**
2	Band-scale entropy (RF, Alpha)	0.05
3	Intra-regional CSE (LC)	0.05
4	BDI score	0.04
5	Inter-regional CSE (RF-RP)	0.04
6	Band-scale entropy (RC, Theta)	0.04
7	Band-scale entropy (RF, Beta)	0.04
8	Band-scale entropy (LF, Beta)	0.03
9	Intra-regional CSE (RF)	0.03
10	Band-scale entropy (RP, Alpha)	0.02
11	Inter-regional CSE (LF-RC)	0.02
12	Intra-regional CSE (RC)	0.02
13	Band-scale entropy (LP, Gamma)	0.02
14	Band-scale entropy (RF, Theta)	0.02
15	Band-scale entropy (RP, Gamma)	0.01

Panel B: Stable MDD vs. converted BD (n = 66), MLP		
Rank	Feature	Mean |SHAP value|
**1**	**Band-scale entropy (LP, Theta)**	**0.04**
2	Inter-regional CSE (LC-RC)	0.03
3	Inter-regional CSE (LF-RP)	0.03
4	Inter-regional CSE (RP-LC)	0.03
5	Inter-regional CSE (RP-RC)	0.02
6	Inter-regional CSE (RF-RP)	0.02
7	Inter-regional CSE (LF-LP)	0.02
8	Inter-regional CSE (LF-RC)	0.02
9	Band-scale entropy (LC, Gamma)	0.01
10	Band-scale entropy (LF, Alpha)	0.01
11	Inter-regional CSE (LF-LC)	0.01
12	Band-scale entropy (LC, Theta)	0.01
13	Intra-regional CSE (RC)	0.01
14	Band-scale entropy (RF, Alpha)	0.01
15	Band-scale entropy (RC, Alpha)	0.01

Panel C: Stable MDD vs. diagnosed BD (n = 76), RBF-SVM		
Rank	Feature	Mean |SHAP value|
**1**	**BDI score**	**0.11**
2	Band-scale entropy (LF, Beta)	0.02
3	Band-scale entropy (RC, Beta)	0.02
4	Intra-regional CSE (RF)	0.01
5	Intra-regional CSE (LC)	0.01
6	Band-scale entropy (LF, Theta)	0.01
7	Band-scale entropy (RP, Theta)	0.01
8	Band-scale entropy (LF, Gamma)	0.01
9	Band-scale entropy (LC, Gamma)	0.01
10	Inter-regional CSE (LF-RF)	0.01
11	Intra-regional CSE (RP)	0.01
12	Band-scale entropy (RC, Theta)	0.01
13	Band-scale entropy (RC, Alpha)	0.01
14	Band-scale entropy (LP, Theta)	0.01
15	Inter-regional CSE (RF-RP)	0.01

**Note.** BDI, Beck Depression Inventory; CSE, cross-sample entropy; LF, left frontal; RF, right frontal; LC, left central; RC, right central; LP, left parietal; RP, right parietal; RBF-SVM, support vector machine with a radial basis function kernel; MLP, multilayer perceptron. BDI was included in Panels A and C and excluded from Panel B.

Navigate back to Figure 5..
