# Supplementary material for: Machine learning–based differentiation of major depressive disorder and bipolar disorder using entropy-derived EEG biomarkers in drug-naïve patients
Source: CNS Spectr. 2026 May 11;31(1):e40. doi: 10.1017/S1092852926100923 (PMC13732613; doi:10.1017/S1092852926100923)
Supplement: Supplementary file 1 [file S1092852926100923sup001.docx]

**Supplementary Material**

*Machine learning-based differentiation of major depressive disorder and bipolar disorder using entropy-derived EEG biomarkers in drug-naïve patients*

**Table of Contents**

**Supplementary Table S1.** Comparison of classifier performance across three binary classification tasks

**Supplementary Table S2.** Comparison of feature selection strategies within the best-performing classifier for each task

**Supplementary Table S3.** Comparison of class-imbalance resampling strategies within the best-performing classifier for each task

**Supplementary Table S1.** Comparison of classifier performance across three binary classification tasks. Each classifier was independently optimized via nested cross-validation (5 folds × 20 repeats) with its best-performing feature selection and resampling combination. Performance is reported as pooled estimate (95% CI). Bold indicates the best-performing classifier for each task.

| **Rank** | **Classifier** | **Feature selection** | **Resampling** | **BA (%)** | **AUC** | **Acc (%)** |
| --- | --- | --- | --- | --- | --- | --- |
| **Task 1: Stable MDD vs. BD spectrum (*n* = 92)** | | | | | | |
| **1** | **SVM (RBF)** | **None (full set)** | **None** | **71.5 (61.8–81.3)** | **0.758 (0.648–0.861)** | **71.7 (62.0–81.5)** |
| 2 | Ridge classifier | None (full set) | None | 69.0 (58.9–78.2) | 0.713 (0.592–0.822) | 69.6 (59.8–78.3) |
| 3 | LDA | None (full set) | None | 68.0 (57.8–77.3) | 0.737 (0.625–0.836) | 68.5 (58.7–78.3) |
| 4 | MLP | RFE | None | 70.0 (60.1–79.4) | 0.691 (0.572–0.801) | 70.7 (60.9–79.3) |
| 5 | SVM (Linear) | None (full set) | None | 68.8 (58.7–77.9) | 0.694 (0.574–0.805) | 69.6 (59.8–78.3) |
| 6 | Random forest | RFE | SMOTE | 66.7 (56.5–76.4) | 0.714 (0.601–0.814) | 66.3 (56.5–76.1) |
| 7 | Logistic regression | None (full set) | SMOTE | 66.8 (56.7–76.3) | 0.698 (0.578–0.809) | 67.4 (57.6–77.2) |
| **Task 2: Stable MDD vs. converted BD (*n* = 66)** | | | | | | |
| **1** | **MLP** | **None (full set)** | **Borderline-SMOTE** | **65.4 (52.7–79.1)** | **0.661 (0.519–0.798)** | **63.6 (53.0–75.8)** |
| 2 | LDA | None (full set) | None | 64.5 (51.5–76.9) | 0.620 (0.466–0.771) | 59.1 (47.0–71.2) |
| 3 | Ridge classifier | None (full set) | None | 58.0 (43.7–71.6) | 0.550 (0.382–0.708) | 62.1 (50.0–72.7) |
| 4 | Random forest | None (full set) | Borderline-SMOTE | 58.1 (45.0–72.0) | 0.575 (0.413–0.743) | 59.1 (48.5–71.2) |
| 5 | SVM (RBF) | None (full set) | SMOTE | 59.2 (45.5–72.7) | 0.547 (0.380–0.707) | 57.6 (45.5–69.7) |
| 6 | SVM (Linear) | None (full set) | None | 55.9 (41.7–70.1) | 0.489 (0.310–0.667) | 62.1 (50.0–72.7) |
| 7 | Logistic regression | RFE | Borderline-SMOTE | 56.0 (41.8–70.2) | 0.485 (0.302–0.664) | 59.1 (47.0–71.2) |
| **Task 3: Stable MDD vs. diagnosed BD (*n* = 76)** | | | | | | |
| **1** | **SVM (RBF)** | **None (full set)** | **SMOTE** | **76.6 (65.9–87.2)** | **0.832 (0.709–0.930)** | **78.9 (69.7–88.2)** |
| 2 | MLP | RFE | SMOTE | 77.6 (66.4–88.0) | 0.799 (0.659–0.915) | 80.3 (71.1–89.5) |
| 3 | Random forest | RFE | SMOTE | 75.5 (64.7–85.6) | 0.810 (0.687–0.909) | 76.3 (67.1–85.5) |
| 4 | Ridge classifier | None (full set) | SMOTE | 75.6 (64.6–86.0) | 0.768 (0.637–0.882) | 77.6 (68.4–86.8) |
| 5 | SVM (Linear) | ANOVA *F*-test | SMOTE | 73.7 (62.5–84.4) | 0.776 (0.651–0.887) | 76.3 (65.8–85.5) |
| 6 | LDA | ANOVA *F*-test | None | 71.8 (60.3–82.5) | 0.802 (0.678–0.905) | 75.0 (64.5–84.2) |
| 7 | Logistic regression | ANOVA *F*-test | Borderline-SMOTE | 72.7 (62.0–83.1) | 0.773 (0.642–0.886) | 75.0 (65.8–84.2) |

*Note.* BA = balanced accuracy; AUC = area under the receiver operating characteristic curve; Acc = overall accuracy; CI = confidence interval. The best-performing classifier for each task was selected based on a composite mean-rank aggregation across six pooled performance metrics (balanced accuracy, AUC, accuracy, sensitivity, specificity, and F1-score), rather than any single metric alone. For each metric, all 63 model–feature selection–resampling combinations were ranked, and the combination with the lowest mean rank was selected as optimal. For Tasks 1 and 3, the EEG + BDI feature configuration was used as the default; for Task 2, only the EEG-only configuration was evaluated (see Section 3.8). Feature configuration comparisons (EEG-only, EEG + BDI, and BDI-only) were conducted separately for the optimal pipeline identified in each task. MDD = major depressive disorder; BD = bipolar disorder; SVM = support vector machine; RBF = radial basis function; MLP = multilayer perceptron; LDA = linear discriminant analysis; RFE = recursive feature elimination; SMOTE = Synthetic Minority Over-sampling Technique.

**Supplementary Table S2.** Comparison of feature selection strategies within the best-performing classifier for each task. For each task, the best-performing classifier and resampling strategy (identified in Supplementary Table S1) were fixed, and each feature selection strategy was evaluated. Performance is reported as pooled estimate (95% CI). Bold indicates the best-performing feature selection strategy for each task.

| **Feature selection** | **Type** | **BA (%)** | **AUC** | **Acc (%)** |
| --- | --- | --- | --- | --- |
| **Task 1: SVM (RBF) + EEG + BDI + None fixed (*n* = 92)** | | | | |
| **None (full set)** | **—** | **71.5 (61.8–81.3)** | **0.758 (0.648–0.861)** | **71.7 (62.0–81.5)** |
| RFE | Wrapper | 73.1 (63.7–82.2) | 0.744 (0.631–0.844) | 72.8 (63.0–81.5) |
| ANOVA *F*-test | Filter | 70.3 (60.1–79.7) | 0.714 (0.596–0.825) | 70.7 (60.9–80.4) |
| **Task 2: MLP + EEG-only + Borderline-SMOTE fixed (*n* = 66)** | | | | |
| **None (full set)** | **—** | **65.4 (52.7–79.1)** | **0.661 (0.519–0.798)** | **63.6 (53.0–75.8)** |
| ANOVA *F*-test | Filter | 60.2 (46.5–74.5) | 0.625 (0.461–0.781) | 59.1 (47.0–71.2) |
| RFE | Wrapper | 52.0 (37.8–66.1) | 0.468 (0.288–0.651) | 53.0 (40.9–65.2) |
| **Task 3: SVM (RBF) + EEG + BDI + SMOTE fixed (*n* = 76)** | | | | |
| **None (full set)** | **—** | **76.6 (65.9–87.2)** | **0.832 (0.709–0.930)** | **78.9 (69.7–88.2)** |
| ANOVA *F*-test | Filter | 72.6 (61.5–83.2) | 0.815 (0.684–0.920) | 73.7 (63.2–82.9) |
| RFE | Wrapper | 68.5 (57.6–79.2) | 0.792 (0.644–0.911) | 65.8 (55.3–76.3) |

*Note.* BA = balanced accuracy; AUC = area under the receiver operating characteristic curve; Acc = overall accuracy; CI = confidence interval; RFE = recursive feature elimination; SVM = support vector machine; RBF = radial basis function; MLP = multilayer perceptron; SMOTE = Synthetic Minority Over-sampling Technique. The ranking of feature selection strategies within each task was determined using mean-rank aggregation across all six pooled metrics (see Supplementary Table S1 note).

**Supplementary Table S3.** Comparison of class-imbalance resampling strategies within the best-performing classifier for each task. For each task, the best-performing classifier and feature selection strategy (identified in Supplementary Table S1) were fixed, and each resampling strategy was evaluated. Performance is reported as pooled estimate (95% CI). Bold indicates the best-performing resampling strategy for each task.

| **Resampling strategy** | **Type** | **BA (%)** | **AUC** | **Acc (%)** |
| --- | --- | --- | --- | --- |
| **Task 1: SVM (RBF) + EEG + BDI + None (full set) fixed (*n* = 92)** | | | | |
| **None** | **—** | **71.5 (61.8–81.3)** | **0.758 (0.648–0.861)** | **71.7 (62.0–81.5)** |
| Borderline-SMOTE | Synthetic over-sampling | 70.3 (60.0–79.9) | 0.767 (0.658–0.866) | 70.7 (60.9–80.4) |
| SMOTE | Synthetic over-sampling | 70.5 (60.6–80.0) | 0.767 (0.657–0.867) | 70.7 (60.9–80.4) |
| **Task 2: MLP + EEG-only + None (full set) fixed (*n* = 66)** | | | | |
| **Borderline-SMOTE** | **Synthetic over-sampling** | **65.4 (52.7–79.1)** | **0.661 (0.519–0.798)** | **63.6 (53.0–75.8)** |
| SMOTE | Synthetic over-sampling | 63.4 (50.3–76.7) | 0.644 (0.505–0.782) | 60.6 (50.0–72.7) |
| None | — | 55.1 (41.4–69.4) | 0.675 (0.542–0.809) | 54.5 (42.4–66.7) |
| **Task 3: SVM (RBF) + EEG + BDI + None (full set) fixed (*n* = 76)** | | | | |
| **SMOTE** | **Synthetic over-sampling** | **76.6 (65.9–87.2)** | **0.832 (0.709–0.930)** | **78.9 (69.7–88.2)** |
| Borderline-SMOTE | Synthetic over-sampling | 76.6 (66.0–86.9) | 0.830 (0.701–0.932) | 78.9 (69.7–88.2) |
| None | — | 73.6 (62.9–84.3) | 0.826 (0.697–0.927) | 75.0 (65.8–84.2) |

*Note.* BA = balanced accuracy; AUC = area under the receiver operating characteristic curve; Acc = overall accuracy; CI = confidence interval; SMOTE = Synthetic Minority Over-sampling Technique; SVM = support vector machine; RBF = radial basis function; MLP = multilayer perceptron; RFE = recursive feature elimination. The ranking of resampling strategies within each task was determined using mean-rank aggregation across all six pooled metrics (see Supplementary Table S1 note).
